# Dedifferentiation and Proliferation of Artery Endothelial Cells Drive Coronary Collateral Development in Mice

**DOI:** 10.1161/ATVBAHA.123.319319

**Published:** 2023-06-22

**Authors:** Gauri Arolkar, Sneha K. Kumar, Hanjay Wang, Karen M. Gonzalez, Suraj Kumar, Bhavnesh Bishnoi, Pamela E. Rios Coronado, Y. Joseph Woo, Kristy Red-Horse, Soumyashree Das

**Affiliations:** National Centre for Biological Sciences, Tata Institute of Fundamental Research, Bengaluru, India (G.A., S.K.K., S.K., B.B., S.D.).; Department of Cardiothoracic Surgery (H.W., Y.J.W.), Stanford University School of Medicine, CA.; Institute for Stem Cell Biology and Regenerative Medicine (K.M.G., K.R.-H.), Stanford University School of Medicine, CA.; Department of Biology (K.M.G., K.R.-H.), Stanford University, CA.; Department of Bioengineering (P.E.R.C.), Stanford University, CA.; Howard Hughes Medical Institute, Chevy Chase, MD (K.R.-H.).

**Keywords:** coronary arteries, endothelial cells, ischemia, myocardial infarction, vascular endothelial growth factor receptor-2

## Abstract

Collateral arteries act as natural bypasses which reroute blood flow to ischemic regions and facilitate tissue regeneration. In an injured heart, neonatal artery endothelial cells orchestrate a systematic series of cellular events, which includes their outward migration, proliferation, and coalescence into fully functional collateral arteries. This process, called artery reassembly, aids complete cardiac regeneration in neonatal hearts but is absent in adults. The reason for this age-dependent disparity in artery cell response is completely unknown. In this study, we investigated if regenerative potential of coronary arteries is dictated by their ability to dedifferentiate.

**Methods:**

Single-cell RNA sequencing of coronary endothelial cells was performed to identify differences in molecular profiles of neonatal and adult endothelial cells in mice. Findings from this in silico analyses were confirmed with in vivo experiments using genetic lineage tracing, whole organ immunostaining, confocal imaging, and cardiac functional assays in mice.

**Results:**

Upon coronary occlusion, neonates showed a significant increase in actively cycling artery cells and expressed prominent dedifferentiation markers. Data from in silico pathway analyses and in vivo experiments suggested that upon myocardial infarction, cell cycle reentry of preexisting neonatal artery cells, the subsequent collateral artery formation, and recovery of cardiac function are dependent on arterial VegfR2 (vascular endothelial growth factor receptor-2). This subpopulation of dedifferentiated and proliferating artery cells was absent in nonregenerative postnatal day 7 or adult hearts.

**Conclusions:**

These data indicate that adult artery endothelial cells fail to drive collateral artery development due to their limited ability to dedifferentiate and proliferate.

**Highlights**

- Neonatal artery endothelial cells reenter cell cycle and express markers for dedifferentiation in response to myocardial infarction.
- Artery cell proliferation and dedifferentiation are minimal in injured adult hearts.
- Postmyocardial infarction neonatal artery cell proliferation is VegfR2 (vascular endothelial growth factor receptor-2) dependent.
- Arterial VegfR2 drives artery reassembly and promotes recovery of cardiac function in injured neonates.

Cardiovascular disease represents 32% of all global deaths (World Health Organization) and its burden is increasing sharply in many new parts of the world. Earlier, deaths due to cardiovascular disease dominated the western world, but currently, at least 58% of all cardiovascular disease deaths occur in Asia (http://ghdx.healthdata.org/gbd-results-tool). The primary cause of cardiovascular disease is coronary artery blockage, and can lead to ischemic heart disease. Despite the deadly outcomes, management of symptoms associated with ischemic heart disease often include invasive procedures, which, many patients are ineligible for. Effective therapeutic approaches targeting complete cardiac regeneration can reduce mortality, and manage symptoms of ischemic heart disease. This can be accomplished by understanding, and subsequently utilizing, the molecular mechanisms associated with different physiological aspects of ischemic heart disease. One such mechanism adapted by the heart is building collateral arteries.

Large collateral arteries enhance perfusion,^1^ vascularize hypoxic area, improve myocardial viability,^2^ and are associated with better survival rate.^3^ Collateral arteries are the arterial segments that connect a healthy arteriole to an occluded/nonperfused arteriole and are sufficiently large to carry adequate blood flow to ischemic regions in need. Data from clinical studies suggest that the presence of native/preexisting coronary collateral arteries present at the onset of myocardial infarction (MI) help limit the infarct size, and improves prognosis.^2,4^ The number of preexisting collateral arteries as well as their capacity to undergo remodeling upon occlusion are the key determinants of tissue recovery.^5^ Genetic factors are proposed to regulate differences in collateral number and their functionality across human races and species.^6,7^ However, a complete map of molecular mechanisms/pathways remains unknown. These molecular mechanisms could be regulated by genetic factors and can be a useful tool to design therapeutic interventions for patients with ischemic heart disease.

Brain, a highly complex organ with low regenerative ability, is known to possess preexisting collateral vessels in its pial layer. In mice, these collateral connections develop at an embryonic stage and provide protection against ischemia in adulthood.^8,9^ Preexisting collateral arteries in mouse hindlimb can grow by 2 distinct processes—arterialization of capillary endothelial cells (cap-ECs)^10^ and lateral expansion/widening of smaller preexisting collateral arteries, that is, arteriogenesis.^11^ Both, in brain and hindlimb, genetic background is a major contributor to the variation in the number of observed collaterals.^12,13^ Specifically, polymorphism in vascular endothelial growth factor a (*Vegfa*) locus is shown to control Vegfa expression and predetermine the extent of collateral artery network in mice.^5,14^ Particularly in hindlimb ischemia model, density of preexisting collateral arteries is regulated via VegfR1 (Vegf receptor-1).^14^ However, whether VegfR1 also controls postischemic coronary collateral development in mice is unknown.

In mice, complete cardiac regeneration is accomplished only when hearts are injured within a neonatal regenerative window (postnatal day [P] 0–P6). Any injury beyond this time point (>P7), limits cardiac regeneration.^15,16^ Previously, we have shown that, in regenerative neonatal mice, coronary collateral arteries are built by the systematic execution of 3 major cellular events driven by preexisting artery ECs (aECs)—migration, proliferation, and coalescence.^17^ aECs expressed CXCR4 (C-X-C motif chemokine receptor 4), a receptor for a chemokine, Cxcl12 (C-X-C motif chemokine ligand 12), which steers the migration of aECs into the watershed. This process, named artery reassembly, involves disassembly of artery tips into single migratory artery cells, followed by their reassembly to build new artery segments (collateral arteries). Interestingly, despite *Cxcl12* expression in adult injured watershed, artery reassembly was absent in these hearts. The precise cause for absence of artery reassembly in adult injured hearts remains unknown.

The age-dependent discrepancy in aEC response triggered by MI, could be because of their distinct molecular profiles.^18–20^ To identify potential molecular drivers, we performed single-cell RNA sequencing of neonatal and adult ECs, at a time point before when collateral arteries appear in the injured mouse hearts. Using both bioinformatics analyses and in vivo experiments, we show that, upon injury, neonatal aECs undergo dedifferentiation and VegfR2-mediated proliferation, which is critical for collateral artery formation by artery reassembly and subsequent cardiac function. Both cellular events, arterial dedifferentiation, and proliferation, were absent in older nonregenerative hearts, post-MI. We hope, in future, these findings will aid strategizing ways to induce collateral arteries and enhance regenerative potential of cardiac tissue, by specifically targeting artery cells.

## METHODS

### Data Availability

Raw sequence files and Cell Ranger processed data are available on GEO (accession ID: GSE210307).

Scripts used for the analysis are available at https://github.com/Snehasrivatsa/scRNA.

The data that support the findings of this study are available from the corresponding author upon reasonable request.

### Tissue Digestion and Single-Cell Sorting

Neonatal and adult mouse hearts were dissected, and atria were removed from each heart. Tissue digestion was performed only with cardiac ventricles. Adult mouse hearts were additionally perfused before harvesting hearts. Approximately 15 to 20 neonatal (for sham and MI each) and ≈10 to 12 adult (for sham and MI each) *Cx40CreER*; *Rosa26^TdTomato^* mice were used for tissue digestion. Post explant, ventricles of the hearts were minced using razor blade and fine forceps and transferred to a tissue digestion buffer. The tissue digestion buffer was composed of 500 U/mL Collagenase IV (catalog number LS004186; Worthington), 1.2 U/mL Dispase (catalog number LS02100; Worthington), 32 U/mL DNase I (catalog number LS002007; Worthington) in commercial grade sterile 1× DPBS with Ca^2+^ and Mg^2+^. Each heart was transferred to ≈300 µL of digestion buffer. Hearts bathing in digestion buffer were incubated in 37 °C shaker for 40 to 45 minutes, with periodic resuspension using micropipettes to break down tissue lumps. Digested tissue was then equilibrated with 4 volumes of 5% FBS prepared in 1× DPBS, gently mixed with a micropipette, and filtered using 40 µm of sterile cell strainer. The filtered tissue digest was spun down for 5 minutes at 400*g* at 4 °C, and the cell pellet was gently dissolved in 5 mL of 3% FBS in 1× DPBS. The resuspended cell pellet was spun down for 5 minutes at 400*g* at 4 °C. Supernatant was discarded and the cell pellet was suspended in ≈500 to 600 µL of 3% FBS in 1×DPBS. These suspended single cells were stained with Ter119-Alexafluor-647 (catalog number 116218, 1:100 dilution; BioLegend) for 30 minutes at 4 °C. Poststaining, cells were washed with and resuspended in fresh 3% FBS/1× DPBS, followed by single-cell sorting with fluorescence-activated cell sorting. DAPI (4′,6-diamidino-2-phenylindole) was added before starting the cell sort. Live single ECs (DAPI^−^ Ter119^−^ TdTomato^+^) were collected, in that order, using BD Fluorescence-Activated Cell Sorting Aria II cell sorter. Single cells were counted, and submitted to Stanford Genome Sequencing Service Center for 10× single-cell V3 library preparation. Sequencing was performed using HiSeq 4000.

### Single-Cell RNA Sequencing Data Analyses

#### Processing of FASTA Files

Illumina reads obtained were processed on Cell Ranger (10× Genomics). To demultiplex the BCL files and convert them to FASTQ files, the *mkfastq* function (a command from the package CellRanger) was used. The reads were aligned to mm10 (mouse genome) and TdTomato sequences with the count function (a command from the package CellRanger).

#### Preprocessing on R

The output files of Cell Ranger were imported into RStudio v4.05 using Seurat v4.03.^21^ This was performed with *Read10x* (within Seurat v4.03) feature by providing path to a folder with zip files of expression matrix, features, and barcodes files. The cells were analyzed for nFeature RNA count (number of genes detected per cell) and percent of mitochondrial RNA using the standard preprocessing mentioned on the Seurat.^21^ More details can be found at https://github.com/Snehasrivatsa/scRNA.

The percentage of mito genes expressed in the cells of our data set were <1; hence, no cells were removed. Based on Q/C violin plot, all cells with >250 nFeature RNA count were analyzed. These cutoffs were determined from quality control analyses of all data sets, which include, subsets of cardiac cells filtered with expression of *Cadherin5*, *Pecam1*, or TdTomato from uninjured and injured neonates and adults (Figures S1A and S2A). We obtained and analyzed the following number of cells from each subset. *Cadherin5*>1: 5432 sham and 2200 MI neonatal cells, *Cadherin5*>1: 6256 sham and 1690 MI adult cells, *Pecam1*>1: 5520 sham and 2152 MI neonatal cells, *Pecam1*>1: 6228 sham and 1774 MI adult cells, TdTomato>0: 5557 sham and 497 MI neonatal cells, TdTomato>0: 3090 sham and 892 MI adult cells.

DoubletFinder, an R package, was used to check whether there were any doublets incorporated in the data set. The analysis showed that 7.5% of all cells were doublets in both sham and MI data sets. These doublets were mostly present in endocardial EC (endo-EC), endothelial to mesenchymal transition (endo-MT), and smooth muscle cell (SMC) clusters that were excluded from the study. Transitioning EC (Trans-EC) a unique cluster expressing marker genes for multiple cell types was considered specifically for the analysis (as the chance of it being a doublet is higher). No doublets were found in MI trans-EC cluster. In the sham trans-EC cluster, there were 3 doublets.

### Normalization, Scaling, and Clustering

Data were log normalized with scale factor set to 10 000, followed by finding variable features with n set to 2000 and finally, scaled with *scaledata* (a command from Surat package to scale and center the gene features in the data set). We focused on artery ECs, by subsetting the data using different markers. *Cadherin5* or *Pecam1* (pan endothelial markers) expression >1 was analyzed. A filter for >1 was used for *Cadherin5* or *Pecam1* to exclude non-ECs expressing these genes at a minimal level. As the cells from both filters echoed the same clustering pattern, we used *Cadherin5* cells for further analyses. The pattern acquired by clustering sham and MI data separately or together (merged) echoed the same patterns. Hence, the sham and MI data were integrated using the *IntegrateData* feature (a command from the Seurat package). Normalization, finding variable features, and scaling data were repeated on the integrated data, using the same settings as above.

We performed principal component analysis and top 30 PCs were computed and stored for the downstream analyses. Clusters for the integrated data were obtained with Louvain algorithm with resolution set to 0.5 (https://satijalab.org/seurat/articles/pbmc3k_tutorial.html). The cells were projected and visualized in 2-dimension using Uniform Manifold Approximation and Projection (UMAP) for Dimension Reduction; parameters were n.neighbors set to 10, dims set to 1:20, spread set to 2, and min.dist set to 0.3. Attributes *group.by* and *split.by* were used to observe sham and MI cells. This was done for optimal spread of cells and best visualization.

TdTomato sequence was aligned with and checked in our data sets. Thus, cells with TdTomato expression >0, were analyzed. Since the MI cell number was 10-fold lesser than that of the sham, data were analyzed by subsetting the merged data based on its orig.ident and reclustering the respective cell populations. The steps used with *Cadherin*>1 cells were used for all clustering steps with TdTomato >0.

For expression of genes, data were subsetted for gene >0, the cell numbers per cluster were obtained using table (object@active.ident).

### Cluster Identification

The communities detected were identified with Violin plots (*StackedVlnPlot* from CellChat v1.1.0 package (https://github.com/sqjin/CellChat), *DotPlots* (a feature of Seurat package to visualize gene expression), and feature plots (Seurat argument). Clusters were identified with established markers for a variety of cardiac cell–specific genes. Cluster identifications were confirmed using *AddModuleScore* feature from Seurat package. A list of cell type–specific, established markers were used for this analysis on *Cadherin5*>1, *Pecam1*>1, and TdTomato>0 data sets in both neonate and adult data sets. Average expression of the listed genes for each cell type was visualized using a feature plot. Neonatal trans-EC cluster did not show unique expression of any known cardiac cell types.

Heatmaps were used to check the variation in expression of dedifferentiation genes across sham and MI data sets. *DoHeatmap* feature from Seurat package was used to plot the genes of interest. Violin plots generated from Seurat package were analyzed by using the *split* attribute.

### Cell Cycle Analysis

Updated G2M and S-phase–specific gene lists (from 2019) on Seurat were used. As the genes were in human gene format, *useMart* function from biomaRt v3.12 (R package) was used to obtain hsapiens_gene_ensembl and mmusculus_gene_ensembl from ensembl database. Homology mapping and gene symbol conversion of genes from 2 gene sets were performed using *getLDS* function. Genes were converted to mouse gene format using *convertHumanGeneList*. Cell cycle analysis was performed using standard cell cycle regression (CellCycleScoring) on Seurat.

### Trajectory Analysis

Monocle3^22^ was used for trajectory analysis to understand possible pseudo time relationship between the cell types. Seurat object split into sham and MI from the integrated data, subsetted for ECs alone, was used for the analysis. *as.cell_data_set* feature from SeuratWrappers was used to convert Seurat object to Monocle3 object. Furthermore, the Monocle3 object was subjected to pseudo time analysis using *learn_graph* and *order_cells* functions (https://cole-trapnell-lab.github.io/monocle3/docs/getting_started/). Results were visualized on UMAPs with the aid of *plot_cells* function.

### RNA Velocity

Spliced and unspliced counts were generated from raw Fastq files using kallisto bustools wrapper *kb* (kb-python v0:27:0, https://www.kallistobus.tools/).^23^ Mouse genomic (DNA) FASTA and GTF annotations were downloaded from Ensembl and a transcriptome index was generated by kallisto using *kb ref* command. *Kb count* command was used to generate the count matrix in the form of h5ad file. The h5ad file was converted into a Seurat object using SeuratDisk (v0.0.0.9019). The preprocessing, normalization, scaling, and clustering were done as described earlier. The RNA velocity maps were generated using Velocyto package (velocyto.R v6).^24^

### Analysis of Differentiation State

R package Cellular (Cyto) Trajectory Reconstruction Analysis using Gene counts and Expression (CytoTRACE) v0.3.3^25^ (https://cytotrace.stanford.edu/) was used for cell differentiation analysis. Cell counts and UMAP embeddings were obtained as data frames from sham and MI clusters separately, for the analysis. A CytoTRACE score was assigned to each cell with the CytoTRACE function, which was visualized on the UMAP embeddings with *plotCytoTRACE* feature. We compared the mean CytoTRACE scores between all EC clusters. To do so, CytoTRACE score for each cell and its cluster number was obtained. The mean CytoTRACE score was then calculated for each cluster. These mean values along with standard deviations were plotted as boxplots using *ggboxplot* feature from ggpubr v0.4.0 package.

### Gene Ontology Analysis

List from *FindAllMarkers* was used to recognize processes that appear on publicly available online tool Gene Ontology Enrichment Analysis and Visualization Tool (GOrilla)^26^ (http://cbl-gorilla.cs.technion.ac.il/). Gene Ontology analyses were performed on MI gene list, with sham genes serving as background. Default settings with a filter of *P*<10^−3^ were used. Processes with significant *P* values (<0.0005), and relevant to our study, were plotted on a bar graph.

### g:Profiler

Pathway analysis was performed using an online tool g:Profiler.^27^ The gene list was obtained for neonatal cluster aEC1 (from merged *Cadherin5*>1 data set), using *FindMarkers* feature, with test.use set to “MAST.” The organism was set to *Mus musculus* and the pathways were obtained from databases like KEGG, reactome, WikiPathways, etc.

### Statistical Analysis on Sequenced Data

Wilcoxon test was performed using *RunPresto* feature from SeuratWrappers package. The statistical test was performed on cycling aECs (cyc-aEC) and aEC1 cells from neonatal *Cadherin5*^+^ MI group. Additionally, the test was performed on *Cadherin5*^+^ cyc-aECs from neonatal and adult MI data sets. For this, the data were merged using the *merge* feature from Seurat and retained as such without any further modifications. Test was done to check statistical significance for expression of *Cx40*, *Cxcr4*, *Aplnr*, *Car4*, *Apln*, *Foxm1, Vegfa*, *Vegfc*, and *Kdr* across different treatments (sham and MI in Figure 4) or clusters (cyc-aEC and aEC1 in neonates, in Figure S7A).

**Figure 1.**
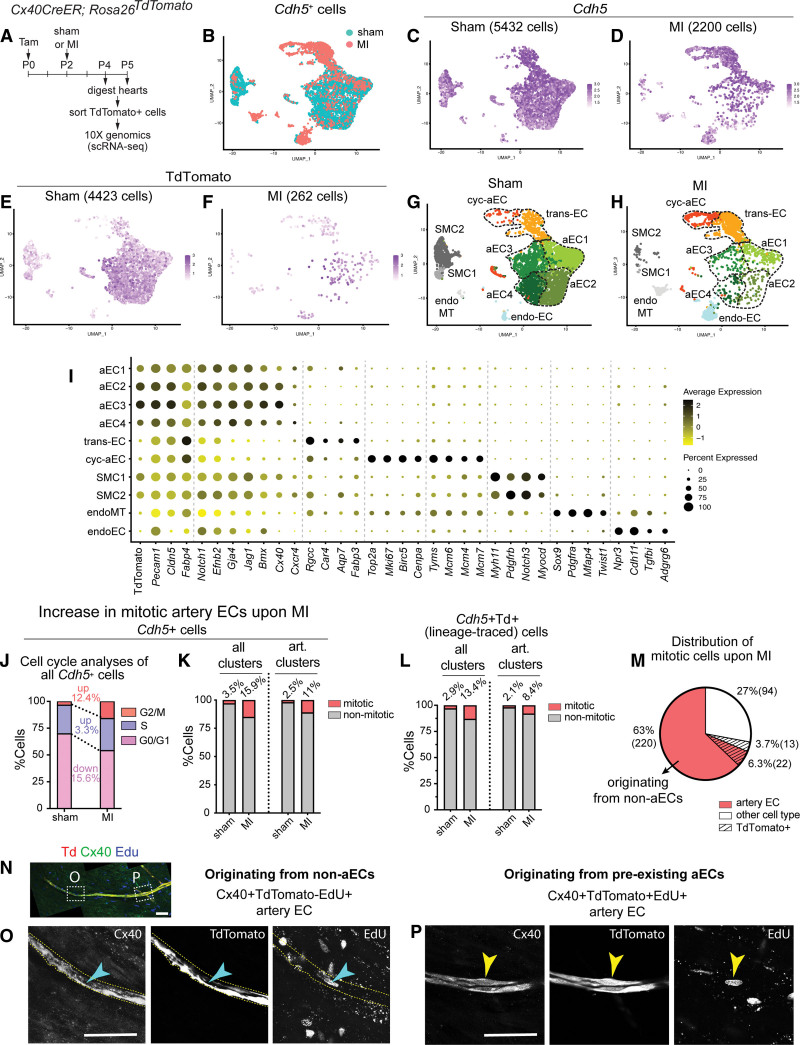
**Single-cell analyses of neonatal artery (art) endothelial cells (aECs). A**, Experimental setup to obtain neonatal cardiac cells for single-cell RNA (scRNA) sequencing using 10× genomics (**B**) Uniform Manifold Approximation and Projection (UMAP) showing the distribution of *Cadherin5*^+^ neonatal sham and myocardial infarction (MI) cells. **C** and **D**, Feature plot showing the distribution of *Cadherin5^+^* cells from (**C**) sham and (**D**) MI group. **E** and **F**, Distribution of TdTomato (Td) expressing cells within *Cadherin5*^+^ (**E**) sham and (**F**) MI group. **G** and **H**, UMAP showing distribution of (**G**) sham and (**H**) MI cell clusters from the merged *Cadherin5*^+^ cells. **I**, Dot plot showing average expression and percentage of cells, expressing relevant markers used for cluster identification. **J**, Graph showing percentage of sham and MI *Cadherin5*^+^ cells in different cell cycle stages based on cell cycle sorting analysis on Seurat. **K**, Graph showing percentage of *Cadherin5*^+^ cells undergoing G2 to M transition, in sham and MI groups, in all clusters or in only aEC clusters (aEC1–4 and cycling aECs [cyc-aEC]). **L**, Graph showing percentage of *Cadherin5*^+^ and *Cx40creER* lineage–traced (Td^+^) cells undergoing G2 to M transition, in sham and MI groups, in all clusters or only aEC clusters (aEC1–4 and cyc-aEC). **M**, Pie chart showing the percent distribution of G2/M *Cadherin5*^+^ cells, upon MI. **N** through **P**, Confocal image of an (**N**) art tip from a post-MI heart, immunostained for Cx40, lineage traced with Td and labeled with 5-ethynyl-2′-deoxyuridine (EdU) show presence of (**O**) Cx40^+^ Td^−^ EdU^+^ (cyan arrowhead) aEC and (**P**) Cx40^+^ Td^+^ EdU^+^ (yellow arrowhead) within the same art branch. Scale bar: **N–P**, 50 µm. endo-EC indicates endocardial EC; endo-MT, endothelial to mesenchymal transition; P, postnatal day; SMC, smooth muscle cell; and Trans-EC, transitioning EC.

**Figure 2.**
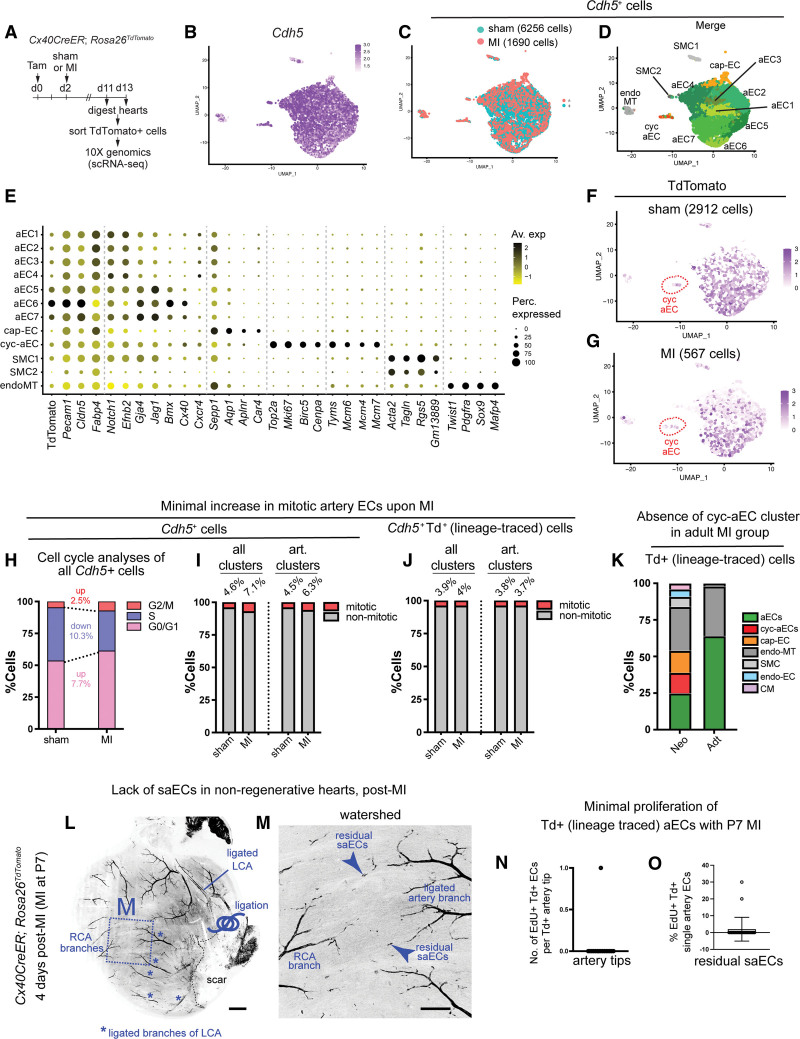
**Cell cycle analyses of adult (Adt) artery (art) endothelial cells (aECs) postmyocardial infarction (MI). A**, Experimental setup to obtain Adt cardiac cells for single-cell RNA (scRNA) sequencing using 10× genomics. **B**, Feature Plot showing the distribution of Adt *Cadherin5^+^* cells from both sham and MI groups. **C**, Uniform Manifold Approximation and Projection (UMAP) showing the distribution of *Cadherin5*^+^ Adt sham and MI cells. **D**, UMAP showing distribution of both sham and MI cell clusters from the merged Adt *Cadherin5*^+^ cells. **E**, DotPlot showing average (av.) expression and percentage of cells, expressing relevant markers used for cluster identification in **D. F** and **G**, Distribution of TdTomato expressing cells within *Cadherin5*^+^ (**F**) sham and (**G**) MI group. **H**, Graph showing percentage of Adt, sham, and MI, *Cadherin5*^+^ cells in different cell cycle stages based on cell sorting analysis on Seurat. **I**, Graph showing percentage of *Cadherin5*^+^ cells undergoing G2 to M transition, in sham and MI groups, in all clusters or in only aEC clusters (aEC1–7 and cycling aECs [cyc-aEC]). **J**, Graph showing percentage of *Cadherin5*^+^ and *Cx40creER* lineage–traced (TdTomato^+^) cells undergoing G2 to M transition, in sham and MI groups, in all clusters or in only aEC clusters (aEC1–7 and cyc-aEC). **K**, Single-cell data analyses of all TdTomato^+^ cells from neonatal (Neo) and Adt MI groups, showing distribution of different cell types that originate from *Cx40CreER* lineage labeling or subsequent tracing. **L** and **M**, Representative image of postnatal day (P) 11 *Cx40CreER*; *Rosa26^TdTomato^* (**L**) whole heart and respective (**M**) watershed region, 4 days post-MI. MI was performed at P7. Lineage-traced aECs are shown in black. *Marks ligated branches of left coronary art (LCA), arrowheads point to residual single aECs (saECs). **N** and **O**, Graph showing number and percentage of 5-ethynyl-2′-deoxyuridine (EdU)^+^ Tdtomato^+^ ECs in (**N**) art tips and (**O**) residual saECs, respectively, at P11. MIs were performed at P7. Scale bars: **L**, 500 µm; **M**, 200 µm. Cap-EC indicates capillary EC; endo-MT, endothelial to mesenchymal transition; RCA, right coronary art; and SMC, smooth muscle cell.

**Figure 3.**
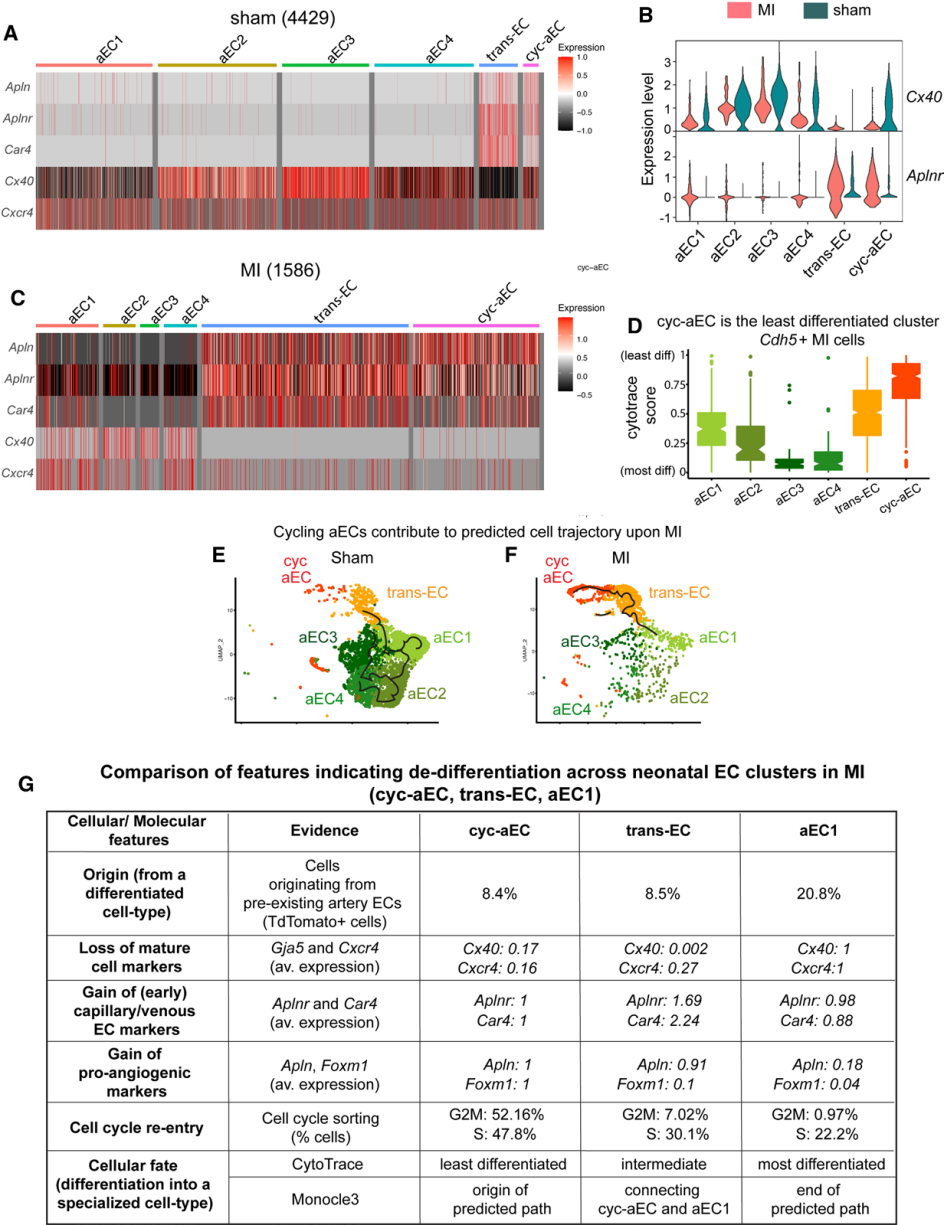
**In silico assessment of dedifferentiation in neonatal cycling artery endothelial cells (aECs). A**, Heatmap showing gene expression of *Apln*, *Aplnr*, *Car4*, *Cx40*, and *Cxcr4* across all *Cadherin5*^+^ clusters in neonatal sham group. **B**, Violin plots comparing expression of *Cx40* and *Aplnr* between different EC clusters in sham and myocardial infarction (MI) group. **C**, Heatmap showing gene expression of *Apln*, *Aplnr*, *Car4*, *Cx40*, and *Cxcr4* across all *Cadherin5*^+^ clusters in neonatal MI group. **D**, Box plots illustrating a relative differentiation state of *Cadherin5*^+^ MI cells with scores obtained from Cellular Trajectory Reconstruction Analysis using Gene counts and Expression. **E** and **F**, Trajectories obtained by performing Pseudo time analysis on neonatal *Cadherin5*^+^ (**E**) sham cells or (**F**) MI cells, using Monocle3. **G**, Comparison of dedifferentiation features across the clusters involved in pseudo time analysis of *Cadherin5*^+^ neonatal MI cells (cycling–aEC [cyc-aEC], transitioning EC [trans-EC], and aEC1).

### Experimental Model

Following mouse lines were used in this study. *Rosa26^TdTomato^* Cre reporter line (The Jackson Laboratory, B6.Cg-Gt[ROSA]26Sortm9[CAG-TdTomato]Hze/J, stock number 007909), *Cx40CreER*,^28^ *VegfR2 flox* (The Jackson Laboratory, *Kdr^tm2Sato^*/J, stock number 018977), *Cx40^eGFP/+^*.^29^

Mice with mixed strain background were used. All mice were housed and bred either at Stanford (in accordance with Institutional Animal Care and Use Committee) or National Centre for Biological Sciences animal facility (in accordance with Institutional Animal Ethics Committee). Mice were fed gamma irradiated vegetal rodent maintenance diet SAFEA40. All tamoxifen injections (6 mg dissolved in corn oil) were administered intraperitoneally to nursing mothers for Cre activation in new born pups. Efficiency of tamoxifen and Cre activation has been shown earlier to be ≈84% using *Cx40CreER* mice and ≈86% using *ApjCreER* mice.^17^ Data shown in this study is compiled from age-matched males and females from several litters. Data from both males and females were combined.

### Experimental Study Design

Sample size for the current experiment was determined from our previous study conducted with the same animal model with similar treatments.^17^ In any given litter, animals were categorized as control or knockout based on their genotype, and irrespective of the sex. Severity of MI was determined by first checking the location of left coronary artery (LCA) ligation under fluorescence stereo-microscope (to check knot around TdTomato^+^ arteries). Hearts with severe MI (where either the main LCA or the 2 primary branches of LCA were captured) were included in this study. Hearts with mild MI (where secondary or tertiary branches were ligated) were excluded from the study. Biological replicates from multiple litters were included in the study and are mentioned below for each in vivo experiment (Table).

**Table.**
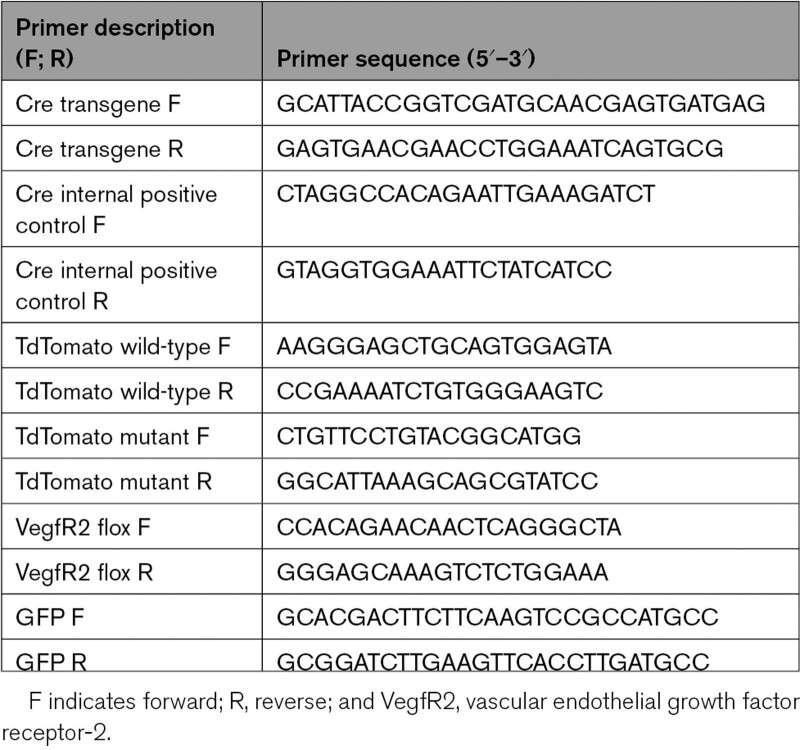
Genotyping Information

### Adult LCA Ligations

Adult mice were anesthetized in a 4% isoflurane chamber and then intubated with a 20G angiocatheter. General anesthesia was maintained with 1.5% to 4% isoflurane during mechanical ventilation at a respiratory rate of 180 breaths per minute. The mice were placed in right lateral decubitus position, and the left chest was shaved and sterilely prepped. 1 mg/kg bupivacaine and 0.5 mg/kg Buprenorphine were injected subcutaneously for perioperative analgesia. A 1 cm left thoracotomy incision was made, and the pericardium was opened. The LCA was identified and permanently ligated using a 6-0 polypropylene suture. The chest was then closed in layers using 5-0 polypropylene sutures. Isoflurane was then weaned off and the mice were extubated after regaining physiological respirations. 5 mg/kg Carprofen was injected subcutaneously daily for the first 3 days after surgery for postoperative analgesia. For adult MI experiments, 2 to 3 months old mice were used (both males and females). To activate Cre, a single dose of 6 mg tamoxifen intraperitoneal injections were administered to each mouse, 2 days before MI. Echocardiograms were obtained from P32/P33 adult mice (sham or MI surgeries at P2) ≈30 days post-MI using VisualSonics Vevo 3100 imaging system (FUJIFILM VisualSonics, Inc) with an MX400 20–46 MHz transducer. Data were analyzed using Vevo LAB Version 3.1.0 (Build 13029).

### Neonatal LCA Ligations

Technical details of the procedure are referred and modified from Mahmoud et al.^17,30^ To induce hypothermic arrest, neonatal mice (P2 or P7) were incubated on ice. Neonates were wrapped with a 3-layer, precooled, gauze piece for ≈3 minutes. Anesthesia was confirmed using toe pinch. Surgery was performed in sterile conditions from start to end, using sterile surgical tools at all times. Animals were placed in a supine position, on a sterile surgical drape, wrapped around cold ice pack. Animals were then prepped with betadine and 70% ethanol. Left anterolateral thoracotomy was performed by making an incision on fourth intercostal space to access heart, under a dissecting microscope. The LCA was identified and ligated by passing a needle attached to 8-0 nonabsorbable prolene suture (catalog number NW3322; Ethicon Ethilon). Post-ligation, the 8-0 prolene suture was used to close the ribs, intercostal muscles, and skin separately. The neonate was then transferred to warm plate (37 °C) to gain consciousness and recover completely. When conscious, neonates were returned to their mother’s cage and closely observed over the next few days.

### Whole Mount Immunostaining of Neonatal Hearts

The procedure has been described earlier.^17^ Neonatal whole hearts were dissected and immediately fixed in 4% paraformaldehyde at 4 °C for 1 hour on a rocker. Hearts were then subjected to 3- and 15-minute washes, with 1× PBS at 4 °C. Primary antibody staining was done by incubating hearts in respective dilutions of primary antibodies, prepared in at least 5 volumes of 1× PBS containing 0.5% Triton-X 100 (0.5% PBT). Incubations were done overnight at 4 °C, on a shaker. Hearts were washed in 20 volumes of 0.5% PBT for 12 hours, with a change in wash buffer (0.5% PBT), every 2 hours at 4 °C. The secondary antibody staining was performed by incubating hearts in 1:250 dilution prepared in 0.5% PBT. Incubation was done on a shaker. Next, hearts were washed in 20 volumes of 0.5% PBT, for 4 days with a change in wash buffer, every 2 hours at 4 °C. All washes were performed with continuous but gentle shaking. Hearts were cleared in at least 2 volumes of anti-fade mounting media (https://www.jacksonimmu number com/technical/products/protocols/anti-fade) for 2 hours, in dark, at room temperature. Whole hearts were then stored at −20 °C till imaged. Poststaining washes were done in 15- and 50-mL tubes, whereas incubation in antibodies or mounting media were performed in 1.5 mL microtubes.

Neonates were injected with 30 µL of 10 mM 5-ethynyl-2′-deoxyuridine (EdU; prepared in 1× commercial grade PBS, catalog number 10010023) for 1 hour, before harvesting their hearts. EdU label detection was combined with postsecondary immunostaining steps as described above and detected using Click-iT staining kit (catalog number C10340; Invitrogen).

### Confocal Imaging of Neonatal Hearts

Whole neonatal (immunostained or genetically labeled/traced with fluorescent reporter) hearts were mounted on a double concave microscope slide (catalog number 7104; Sail Brand) using a microscope coverslip (0.13–0.17 mm, catalog number 48367-059; Thermo Scientific) so that the ventral watershed regions spanning left and right coronary arteries can be imaged. Imaging was performed using Olympus FV3000 upright confocal microscope. Whole hearts were imaged using ×4, ×10, ×20, or ×60 (oil) objectives. Multiple z stacks were acquired using FV31S-SW software. Multiple images of the whole heart were processed on Fiji-ImageJ software, and stitched with Adobe Photoshop.

### Antibodies

Primary antibodies: Anti-CX40 (1:500, catalog number CX40-A, RRID: AB_1616128; Alpha Diagnostics Int, Inc), Anti-VEGFR2 (1:125, catalog number AF644, RRID: AB_355500; R&D Systems). Secondary antibodies: Alexa fluor-conjugated antibodies (488, 555, and 633) from Invitrogen were used at 1:250 dilutions.

### Quantification From Images

#### Collateral Number

Number of *Cx40CreER* lineage–traced (TdTomato positive) collateral arteries were quantified from images acquired using ×10 objective from whole hearts. Number of stacks (12–14) and step size (2 µm) of the images used, were consistent across genotypes. Data were compiled from 6 to 9 litters from each genotype. Eight control, 5 heterozygous, and 14 knockout hearts were used for quantification. One-way ANOVA was performed to check statistical significance.

### Cx40 Intensity in Proliferating aECs

Proliferating TdTomato^+^ Cx40^+^ cardiac aECs (4 days post-MI) were identified with EdU^+^ nuclear labeling. Cytoplasmic intensity of Cx40 in these (TdTomato^+^ Cx40^+^ EdU^+^) ECs was quantified using images acquired with ×60 (oil) objective. Mean fluorescent intensity of cytoplasmic Cx40 signal was measured using a selection tool available with Fiji-ImageJ software. Quantification was performed with 68 single TdTomato^+^ Cx40^+^ EdU^+^ aECs, found in the watershed of 4 hearts, collected 4 days post-MI. Comparison between proliferative (EdU^+^) and nonproliferative (EdU^−^) aECs were made within the same field of view. A paired *t* test was done to check statistical significance. Similar analysis was done on 67 aECs within artery tips or integrated into mature arteries, as identified by Cx40 immunostaining. Data were compiled from 5 MI hearts from different 4 litters.

### Single aEC Proliferation in Control and *VegfR2* Knockout Hearts

EdU^+^ TdTomato^+^ single aECs in the watershed regions were quantified using images acquired with ×20 objective. On an average 20 to 30 TdTomato^+^ single aECs were observed per watershed (each field of view), which was consistent across genotypes. Quantification was performed on 6 control (36 field of view) and 5 *VegfR2* knockout (24 field of view) hearts. A total of 784 control TdTomato^+^ aECs and 512 *VegfR2* knockout TdTomato^+^ aECs were counted for this quantification. Data were compiled from 3 to 5 litters from each genotype. Six control and 5 knockout hearts were used for quantification. An unpaired *t* test was performed to check statistical significance.

### Single aEC Proliferation in P11 MI Hearts (MI at P7)

EdU^+^ TdTomato^+^ proliferating aECs were counted in artery tips and single aECs (saECs) from 3 *Cx40CreER*; *Rosa26^TdTomato^* hearts. ≈80 to 120 µm of distal ends of arteries were identified as artery tips. Twenty-five fields of view from P11 watershed regions, from images taken with ×60 (oil) objective, were used to count a total of 113 TdTomato^+^ residual single cells.

### TdTomato Fluorescence in Control and *VegfR2* Knockout Watershed

Mean fluorescence intensity was measured using Fiji for *Cx40CreER* lineage–traced TdTomato^+^ single aECs found in watershed area 4 days post-MI. Images of watershed regions (each field of view) lacking any large or collateral arteries were acquired with ×20 objective. Watershed images only with single artery cells were considered for this quantification. 3 hearts from each genotype were used for this quantification. An unpaired *t* test was performed to check statistical significance.

### LCA Width

LCA width was measured using a line selection tool on Fiji. The analysis was done for 3 control and 2 *VegfR2* knockout hearts. Width was measured for the main branch of LCA in all hearts and genotypes. An unpaired *t* test was performed to check statistical significance.

## RESULTS

### Increase in Cycling Neonatal aECs, Post-MI

Molecular profiling of cardiac ECs that participate in collateral artery formation can improve our understanding of ways in which complete cardiac reperfusion and regeneration can be attained. We, thus, performed single-cell RNA sequencing of neonatal post-MI heart cells. Specifically, *Cx40CreER; Rosa26^TdTomato^* mice were used, where a single dose of tamoxifen to the lactating mothers induced expression of TdTomato fluorescence in *Cx40* expressing aECs in neonates. Neonates were then subjected to either sham or MI surgeries at P2, and hearts were harvested at P4 or P5 (Figure 1A). TdTomato^+^ cardiac cells were sorted using fluorescence-activated cell sorting method, followed by sequencing using ×10 genomics platform. Sequences were processed using Cell Ranger, and the subsequent output files were analyzed using Seurat on RStudio.^21^

Sorting TdTomato^+^ cells during fluorescence-activated cell sorting, enriched our samples for *Cx40CreER*^+^ lineage-labeled/traced aECs. When analyzed, these TdTomato+ cells were additionally identified to be cardiomyocytes, SMCs, and cells undergoing endo-MT (data not shown). We also obtained some TdTomato^−^ nonartery cells. To specifically study EC biology postinjury, we thus analyzed *Cadherin5*^+^ ECs, which included, both, TdTomato^+^ aECs, genetically labeled between P0 and P2 (Figure 1A), and TdTomato^−^ aECs (if any), identified by expression of known arterial EC genes. Preprocessing and quality control steps (Figure S1A) resulted in 5432 sham and 2200 MI ECs (Figure 1B through 1D), out of which 4423 sham, and 262 MI ECs, expressed at least 1 copy of TdTomato (Figure 1E and 1F). As previously observed,^31,32^ compared with sham, we obtained fewer cells upon MI. This is likely due to extensive cell death following coronary ligation and subsequent tissue ischemia.

Unbiased grouping of *Cadherin5*^+^ ECs from sham and MI resulted in 10 distinct clusters (Figure 1G and 1H). Merged data from sham and MI were projected onto 2-dimensional space using UMAP. The identified cell types were—mature aECs (aEC1–4), transitioning ECs, cyc-aEC, cells undergoing endo-MT, SMC1,2, and endo-EC (Figure 1G and 1H).

We identified the cell types with known vascular and cardiac cell markers and their relative expression levels (Figure 1I; Figures S1 through S3). aEC1–4 expressed markers for mature aECs (*Cx40*, *Cxcr4*) and SMC1,2 expressed markers for both pericytes and SMCs (*Pdgfrb*, *Notch3*). Cells undergoing endo-MT were identified by upregulated expression of mesenchymal markers such as *Col1a1* and *Cdh2* and endo-MT–associated transcription factors like *Serpine1*, *Twist1*, and *Snai1* (Figure 1I; Figure S1B). ECs have been reported to express perivascular markers, such as αSMA (α-smooth muscle actin) and Tagln.^33–38^ Thus, it was not surprising to find that some of these markers were coexpressed by SMC clusters (Figure S1B). An EC cluster expressed several genes specific to G2/M (*Top2a*, *Mki67*, *Birc5*, *Cenpa*, *Kif2c*, *Bub1*, *Nuf2*, and *Hmmr*; Figure 1I; Figure S1C through S1F) and G1/S (*Tyms*, *Mcm4*, *Mcm6*, *Mcm7*, *Ung*, *Chaf1b*, *Fen1*, and *Gmnn*; Figure 1I; Figure S1G through S1J) transition. Sham cells in this particular cluster mostly expressed genes like *Cx40*, *Gja4*, *Jag1*, and *Cxcr4* (Figure S2) known to be significantly expressed and being specific to mouse^39^ and human^40^ coronary arteries. The top 20 genes in this cluster included artery-specific genes^41^ like *Fgfr3*, *Slc45a4*, *Gja4*, and *Ssu2* (data not shown). We hence, named the cluster cyc-aEC. Endo-EC expressed *Npr3*,^42^ *Cdh11*, *Tgfbi*,^43^ and *Adgrg6*^44^ (Figure 1I) and some venous markers (Figure S1K). Nonendothelial cardiac cells were mostly absent in our data set (Figure S1K).

We also identified a cluster of cells (named trans-EC in Figure 1G and 1H) that did not express markers for any single EC subtype. Reclustering this group resulted in 4 distinct EC subclusters, those highly expressed markers for arteries, capillaries, venous/capillaries, and myofibrillar genes^45^ (Figure S3A and S3B). This cluster especially was checked for doublets. No doublets were present in MI trans-EC cluster (data not shown). Trans-EC cluster in the sham data set had 3 doublets (data not shown). 42.8% of artery-like ECs, in this cluster, were TdTomato^+^ (Figure S3C) indicating their origin from preexisting aECs, those labeled between P0 and P2 (Figure 1A). Additionally, this artery-like EC subcluster had fewer cells expressing *Cx40* or *Cxcr4* (Figure S3B). The indistinct molecular nature of this entire cluster, and its proximity to both, a proliferative (cyc-aEC) and mature (aEC1/3) aEC clusters (Figure 1G and 1H), led us to hypothesize that cells in this cluster are in an intermediate state, that is, between a proliferative and mature state. We, hence, called this cluster, trans-EC. Bioinformatics analyses of *Pecam1*^+^ cells, instead of *Cadherin5*^+^ cells, resulted in identification of the same cell types, clustered in a similar manner (data not shown).

Artery cells rarely proliferate, and exiting cell cycle is required for artery cell specification.^17,46–48^ However, we identified an aEC cluster (cyc-aEC) in our data set, expressing several genes that mark G1/S and G2/M transitions (Figure 1I; Figure S1C through S1F). Cell cycle analyses of *Cadherin5^+^* cells showed a 12.4% increase in MI cells undergoing G2 to M transition as compared to the sham group (Figure 1J). The number of MI cells in S-phase increased by 3.3% and G0/G1 phase reduced by 15.6% (Figure 1J), suggesting a higher number of MI cells were likely mitotic.

We next performed cluster-wise quantification of mitotic cells (expressing G2/M markers), in sham and MI groups. In sham group, 2.5% of all mitotic cells were present in artery clusters (aEC1–4 and cyc-aEC; Figure 1K). In MI group, we observed 11% of all mitotic cells in artery clusters (Figure 1K), indicating a 4.4-fold increase in mitotic aECs, upon MI. 15.9% of all *Cadherin5*^+^ cells were mitotic in the MI group, and majority of these cells (≈69.2%) were aECs (Figure 1K), a stably differentiated cell type in neonatal hearts.

### MI Induces New Artery Development, From Sources Independent of Preexisting aECs

Post-MI aEC proliferation is associated with artery reassembly and complete cardiac regeneration.^17^ Use of aEC-specific *Cx40CreER* mouse line, allowed us to mark the lineage of preexisting aECs with TdTomato fluorescent reporter (Figure 1A). We performed cell cycle analysis and quantified the number of *Cadherin5*^+^TdTomato^+^ ECs that expressed genes for G2/M transition and were likely to be mitotic. 13.4% of all *Cadherin5*^+^TdTomato^+^ MI cells were mitotic, ≈63% of which were present in aEC clusters (aEC1–4 and cyc-aEC; Figure 1L). Analyzing mitotic cell distribution within the MI group showed 69.3% of all mitotic cells to be present in artery clusters but very few (6.3%) of them were TdTomato^+^ aECs (Figure 1M). This suggests that MI induces expansion of an aEC population, not lineage labeled by, or traced from *Cx40CreER^+^* cells. This is consistent with our previous observation that injury causes organ-wide artery growth (especially, artery tips), independent of artery reassembly, and through capillary differentiation.^17^

We next performed in vivo experiments to confirm if an aEC population, originating from nonartery sources (as seen in Figure 1M) is indeed proliferating, upon MI. EdU labeled P6 hearts from *Cx40CreER; Rosa26^TdTomato^* neonates (tamoxifen at P0, MI at P2) were harvested and immunostained for Cx40. Cx40 immunolabelled all mature aECs, including *Cx40CreER* lineage–traced TdTomato^+^ preexisting aECs. We indeed, observed numerous Cx40^+^TdTomato^−^ arteries (Figure S4A through S4C), especially in the ischemic regions. Furthermore, we identified these aECs to be proliferating (EdU^+^) and integrated into Cx40^+^ vessels (Figure 1N and 1O; Figure S4A through S4C). As expected, we also observed Cx40^+^TdTomato^+^EdU^+^ aECs (Figure 1N and 1P; Figure S4D and S4E). Thus, ischemia can induce formation of new coronary arteries via multiple mechanisms (Figure S4F). First, coronary collateral artery segments are generated via artery reassembly, a process driven by preexisting aECs. Second, an overall organ-wide artery growth such as extension of artery tips occurs by 2 processes: (1) incorporation of capillary ECs as observed earlier^17^ (also suggested in Figure S4A through S4C) and by (2) proliferation of preexisting (TdTomato^+^) aECs (as shown in Figure S4D and S4E).

### Adult aECs Fail to Proliferate During MI-Induced Artery Neogenesis

New collateral arteries in adult mice, form in response to coronary occlusion within a week of injury.^49^ However, adult coronary collateral arteries are fewer in number, have smaller diameters, and are predicted to perfuse poorly than those in neonate.^50^ The reason for this age-dependent difference in which aECs respond to ischemia is unclear. One possibility could be that adult collateral arteries do not form naturally through artery reassembly. Keeping this in mind, we investigated the molecular nature of adult coronary aECs, and their derivative cell types, in sham and MI mice.

Adult hearts execute artery reassembly only in response to exogeneous Cxcl12. As a result, aEC lineage–traced coronary collateral arteries are observed only after 14 days post-MI.^17^ This is also the time point when the number of adult coronary collateral arteries peak.^39^ To capture single aEC response that precedes new collateral formation, we harvested adult hearts 9 and 11 days post-MI. This stage could reveal adult molecular events, different from that of neonates’. We performed single-cell analyses of cardiac cells sorted from adult *Cx40CreER; Rosa26^TdTomato^* ventricles (Figure 2A; Figure S5). Similar to neonatal data, we obtained both, TdTomato^+^ and some unlabeled cells. After performing quality control steps (Figure S5A), we analyzed 6256 *Cadherin5*^+^ sham and 1690 *Cadherin5*^+^ MI cells (Figure 2B and 2C). Like neonatal data, our adult data set was enriched for aECs (Figure 2D and 2E) and we obtained fewer *Cadherin5*^+^ (Figure 2C) and TdTomato^+^ (Figure 2F and 2G) cells in MI group than sham.

Based on cell-specific gene expression patterns, we obtained 12 adult cell populations (Figure 2D); 7 of which, constituted mature aEC1 to 7, 1 cyc-aEC, 1 capillary EC (cap-EC), 1 endo-MT, and 2 SMC clusters (Figure 2D and 2E). Unlike neonates, trans-EC cluster was absent in adults. Instead, we identified a prominent adult capillary EC population, which when further clustered generated 3 distinct microvascular EC populations—artery-cap-EC, cap-EC, and venous-cap-EC (Figure S5B through S5D). A significant number of art-cap-EC cluster (34%) expressed TdTomato fluorescence (Figure S5D), indicating an origin from preexisting mature arteries. Identity of cycling aECs was confirmed with comparable expression of artery-specific genes (*Gja4*, *Cx40*, *Cxcr4*, *Jag1*) in cyc-aEC and aEC1 to 7 (Figure S5E). A similar cell type distribution was also observed when bioinformatics analyses of *Pecam1*^+^ cells, instead of *Cadherin5^+^* cells, were performed (data not shown).

Adult cardiomyocytes have limited proliferation capacity.^16^ To assess whether the same holds true for cardiac ECs, we performed cell cycle analyses on sham and MI adult *Cadherin5*^+^ ECs. Unlike neonates, we observed only a minimal increase of 2.5% in the number of ECs undergoing G2 to M transition, upon MI (Figure 2H). Further cluster-wise analyses revealed only 1.8% increase in the mitotic (G2/M) aECs (4.5%–6.3%; Figure 2I), compared with 8.5% increase observed in neonates (Figure 1K), upon MI. 4.6% of adult sham cells were mitotic (Figure 2I), suggesting that the preinjury mitotic baseline for adult *Cadherin5*^+^ aECs was higher than that observed in neonates (2.5%; Figure 1K). Thus, in comparison to neonates, fewer adult aECs undergo G2/M transition in response to MI.

P7 or older hearts are nonregenerative^15,16^ and do not illicit artery reassembly.^17^ Cell cycle analyses of *Cadherin5*^+^ TdTomato^+^ lineage–traced adult ECs revealed minimal changes in G2/M ECs including in aEC clusters (aEC1–7, cyc-aEC; Figure 2J). Furthermore, we checked which cell types originate from preexisting aECs, upon MI. For this, we analyzed all TdTomato^+^ cells (labeled and traced from *Cx40CreER*^+^ resident aECs) in adult MI data set and compared it with neonatal MI data set (Figure 2K). Although the neonatal MI group showed a distinct cyc-aEC cluster, this population was absent in the adult MI group (Figure 2K; Figure S5F and S5G). Thus, adult hearts lack a proliferating aEC subpopulation, known to be crucial for artery reassembly, and complete cardiac regeneration.^17^

We next performed in vivo experiments to check if lack of proliferating artery cell population in injured adult hearts (Figure 2J and 2K), is also observed in nonregenerative P7 hearts. *Cx40CreER; Rosa26^TdTomato^* neonates were administered with tamoxifen to lineage label aECs at P5, through P7, followed by MI at P7 (Figure 2L). Proliferation status of *Cx40CreER^+^* lineage–traced, TdTomato^+^, aECs were analyzed with EdU label, before harvesting hearts at P11, 4 days post-MI. As shown earlier,^17^ these hearts did not demonstrate artery reassembly (Figure 2L and 2M), which was evident from lack of lineage-traced (TdTomato^+^) collateral arteries and single aECs in the injured watershed regions (Figure 2M). Very few single aECs were observed adjacent to artery tips (arrowheads, Figure 2M); we called them residual single aECs. EdU^+^ cells in TdTomato^+^ aEC population—in both, artery tips (Figure 2N) and residual single aECs in the watershed (Figure 2O)—were rarely observed. Together, data from both bioinformatics analyses and in vivo experiments indicate that aECs in nonregenerative P7 or adult hearts have limited self-renewing capacities.

### Neonatal aECs Dedifferentiate Upon MI

Stably differentiated cells may attain a less differentiated state and reenter cell cycle in response to pathophysiological cues. The acquired proliferative abilities allow them to transform into less specialized cell type. They can then, either re-differentiate into cells within the same lineage or into cells of a different lineage.^51^ This phenomenon can often be detected through changes in gene expression patterns. Presence of an actively cycling differentiated cell cluster in neonates (cyc-aEC) led us to hypothesize that cells in this cluster dedifferentiate and contribute to new arteries, in response to MI.

Dedifferentiation is indicated by loss of differentiation markers, cell cycle reentry, and expression of early genes, suggesting lack of cell fate specification. We checked whether MI cells when compared with sham cells, manifested any of these dedifferentiation indicators. In sham, *Cx40*, a mature aEC marker, was expressed by cyc-aECs, at levels comparable to mature artery clusters—aEC1 to 4 (Figure 3A and 3B). In MI group, cells in cyc-aEC cluster expressed *Cx40*, at levels much lower than what was observed in sham cyc-aEC (Figure 3B) or the rest of the aEC1 to 4 (Figure 3B and 3C). The reduction of average expression levels of *Cx40* from mature aECs to cycling aECs was of higher magnitude in the MI group (21.6-fold reduced) than in sham (1.3-fold reduced). The percentage of cells expressing *Cx40*, upon MI, reduced in all clusters (Figure 3A and 3C), but this decrease was most prominent in trans-EC (19% in sham, 0% in MI) and cyc-aEC (57% in sham, 3% in MI) clusters (Figure S6A). A similar reduction in number of *Cxcr4*^+^ MI cells was observed specifically in cyc-aEC and trans-EC clusters (Figure S6A). The average expression level of *Cxcr4*, in MI group, reduced by 3.83-fold in trans-EC and 6.7-fold in cyc-aEC, when compared with mature aECs (aEC1–4). This change was negligible in sham group (≈1.1-fold decrease). Thus, there is an overall reduction in number of cells expressing mature artery markers, and their expression levels, in cyc-aEC and trans-EC cluster, upon MI.

During development, an immature coronary vessel plexus undergoes morphogenesis to build coronary arteries.^52^ We hypothesized that cyc-aECs, if dedifferentiating, would reexpress some early genes expressed by this vessel plexus that mostly consists of venous/capillary ECs and from which coronaries originate during development. We, hence, analyzed sham and MI cells for cardiac microvascular genes, such as *Aplnr*^53^ and *Car4*.^40,54^ The number of cells expressing the gene *Aplnr* was increased upon MI (Figure S6A), but the average expression of *AplnR* across these cells was considerably low in mature aECs (aEC1–4) as compared to cyc-aECs or trans-ECs (Figure 3B). *Car4*, also followed the same pattern (Figure 3A and 3C; Figure S6A), indicating both cyc-aEC and trans-EC express venous-capillary marker genes, upon injury.

Angiogenic properties of ECs facilitate vascular remodeling and subsequent cardiac regeneration. We next investigated if cyc-aEC (or trans-EC) clusters expressed any proangiogenic genes, yet another cellular feature of dedifferentiation. Upon MI, 61% of the cyc-aECs and 47% of trans-ECs expressed proangiogenic *Apln* gene (Figure 3A and 3C; Figure S6A), known to drive vascular development^55,56^ and regeneration^57–59^ in vivo. Expression of other angiogenic genes like *Foxm1*,^60,61^ *Nrp2*,^41^ *Col15a1*,^41^ and *Cd34* were enriched in these 2 clusters (Figure S6B through S6E). Additionally, ECs in cyc-aEC and trans-EC clusters expressed various capillary-like/venous specific genes, such as *Gpihbp1*, *Fabp3*, and *Pcdh17* (Figure S6F through S6H). All changes in expression of *Cxcr4*, *Cx40*, *Aplnr*, *Car4*, *Apln* in cyc-aEC between sham and MI groups were found to be statistically significant using Wilcoxon sum-rank test (Table S1). Thus, cyc-aEC and trans-EC clusters are likely to possess angiogenic properties, which are absent in stably differentiated aECs.

Cardiac vascular cells (ECs and SMCs) and cardiomyocytes have common precursor cells. In addition to classic angiogenic EC markers, cyc-aEC, and trans-EC cells also expressed cardiac myofibrillar genes (*Tnnt2*, *Myl3*, and *Myh6*; Figure S5I through S5K). Thus, cardiac ECs maintain low, but active transcription of these cardiomyocyte-specific markers as observed earlier.^45^

The differentiation status of cells and the projected pseudotime, together, can determine the root of differentiation and hence can predict the direction of path taken by cell populations.^25^ A package in RStudio *−* CytoTRACE,^25^ uses the number of genes and their differential expression, to determine relative differentiation status of each cell with respect to others. CytoTRACE score for cycling aEC, in MI data set, was highest among all clusters, making it the least differentiated cluster (Figure 3D). We also performed pseudotime analysis of sham and MI cells using monocle3 package in RStudio. In sham, the predicted trajectory connected trans-EC to aEC1 to 4. Sham cyc-aECs were excluded from the projected path (Figure 3E). In contrast to sham, in MI, cyc-aEC, trans-EC, and aEC1 clusters were associated with the predicted path (Figure 3F). If multiple clusters are connected through a single trajectory, it is likely that, a less specialized cluster differentiates into a terminal cell type. This prediction is supported by our observations from genetic lineage tracing experiments in vivo, where upon MI, *Cx40CreER* lineage-labeled, proliferating aECs give rise to mature coronary collateral arteries^17^ and contribute to organ-wide artery growth (Figure S4F).

We next assessed the relative differentiation status of the 3 clusters participating in the trajectory obtained for MI cells (Figure 3F)—cyc-aEC, tans-EC, and aEC1. We compared the cellular and molecular features indicating dedifferentiation, across these 3 clusters (Figure 3G). As expected, cyc-aEC and aEC1 were the least and most differentiated clusters, respectively (Figure 3G). Trans-EC cluster showed dedifferentiation features intermediate between cyc-aEC and aEC1 (Figure 3G). Thus, it is likely that proliferating (less specialized) aECs in cyc-aEC cluster are differentiating into mature aECs in aEC1 (Figure 3F and 3G). These results were supported by RNA velocity analyses, which, additionally predict directionality of projected trajectories based on the ratio of spliced and unspliced transcripts (Figure S7A and S7B). Together, we hypothesized that upon neonatal MI, at least a subpopulation of mature aECs originates from dedifferentiation and proliferation of preexisting/resident artery cells.

**Figure 4.**
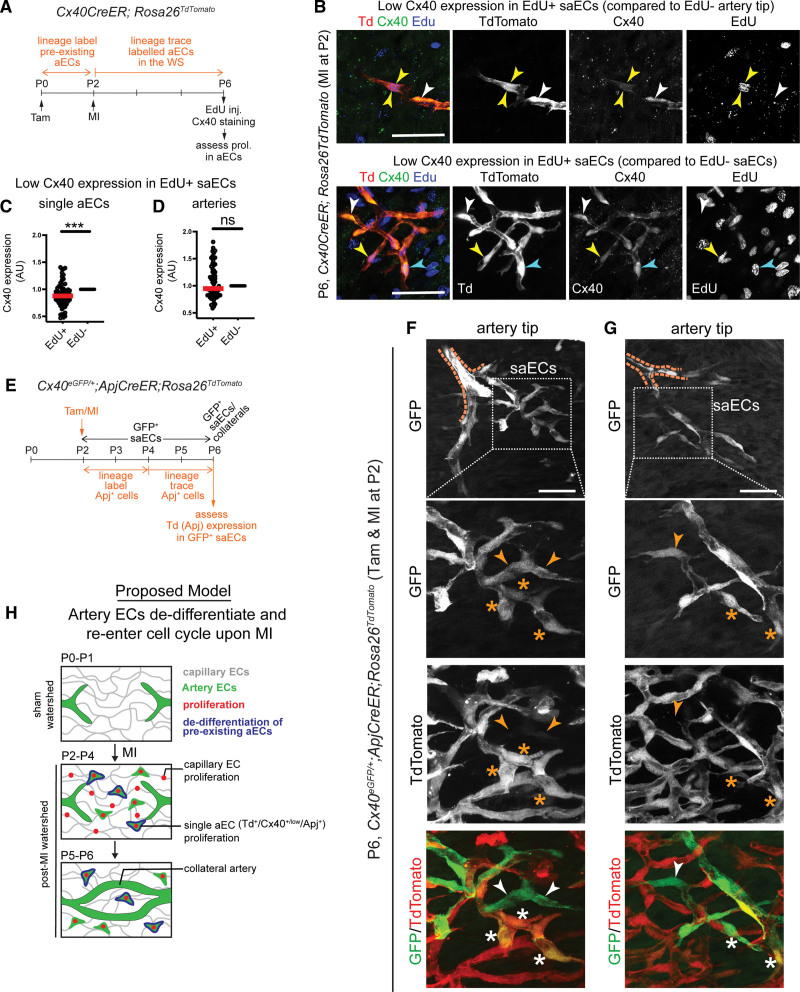
**In vivo assessment of dedifferentiation in neonatal cycling artery endothelial cells (aECs). A**, Experimental setup to check Cx40 levels in proliferating TdTomato aECs postmyocardial infarction (MI; **B**) Confocal images of 5-ethynyl-2′-deoxyuridine (EdU)^+^ TdTomato^+^ single aECs (saEC; yellow arrowhead) and EdU^−^TdTomato^+^ artery tip EC or saECs (white arrowheads) in postnatal day (P) 6 *Cx40CreER*; *Rosa26^TdTomato^* hearts, 4 days post-MI. Cyan arrowhead points to EdU^+^ Tdtomato^+^ saECs with relatively high Cx40 expression. **C** and **D**, Quantification of cytoplasmic Cx40 in P6, *Cx40CreER* lineage–traced (**C**) saECs (*P*=0.0001) and (**D**) aECs (*P*=0.09), 4 days post-MI. **E**. Experimental setup to check presence of *Apj* in TdTomato saECs post-MI. **F** and **G**, Confocal images of GFP+ artery tips and saECs showing presence (marked by *) or absence (marked by arrowhead) of TdTomato+ Apj lineage. **H**, A proposed model whereupon MI, preexisting stably differentiated aECs (green) exit artery tips as saECs, dedifferentiate (blue), proliferate (red), and coalesce into coronary collateral arteries. In our in vivo experiments, saECs are lineage traced from preexisting aECs (TdTomato^+^), but downregulate expression of mature artery marker Cx40 and upregulate cap/vein EC marker Apj upon dedifferentiation. Capillary ECs are shown in gray. Scale bar: **B**, 50 µm; **F–G**, 50 µm.

We checked if aECs, which enter cell cycle upon MI (as seen in our single-cell analyses in Figure 1M), downregulate Cx40 protein expression, in an in vivo setting (Figure 4A). We compared Cx40 protein levels in EdU^+^ and EdU^−^ aECs labeled with Cx40 immunostaining in *Cx40CreER; Rosa26^TdTomato^* neonates, 4 days post-MI (Figure 4B through 4D). Both categories of artery cells, single and associated with lumenized artery vessels, were analyzed. Single aECs observed in ischemic watershed are derived from resident arteries and contribute to artery reassembly. We observed that Cx40 staining was significantly reduced in these EdU^+^ TdTomato^+^ proliferative single aECs (Figure 4B and 4C) when compared with nonproliferative single aECs or artery tips (Figure 4B). In contrast, EdU^+^ proliferating aECs, associated with well-formed arteries, did not show any significant change in their Cx40 expression (Figure 4D). Together, this suggests that Cx40 is downregulated in proliferating single aECs, upon MI.

We further checked if MI-induced single aECs express early markers such as *Apj*, which labels capillary/venous ECs from which arteries originate during coronary development via arterialization. We used *Cx40^eGFP/+^*; *ApjCreER*; *Rosa26^TdTomato^* mice to label arteries with GFP and capillary/venous ECs with TdTomato upon tamoxifen administration. Transgenic neonates were subjected to tamoxifen and MI at P2, to lineage label and trace *ApjCreER*^+^ cells (Figure 4E). Hearts were harvested at P6 when both single aECs and coronary collateral arteries are present (Figure 4E) and analyzed for Apj expression in preexisting aECs, upon MI. We identified single artery cells based on their location, that is, proximity to distal branches of arteries spanning the ischemic watershed area and their morphology, that is, single (not incorporated into any lumenized artery vessels) and spindle-shaped as observed earlier with *Cx40CreER*-lineage trace (Figure 4B). Indeed, many GFP^+^ single aECs expressed TdTomato, indicative of Apj expression at 4 days post-MI (Figure 4F and 4G). These GFP^+^ ECs were not only restricted to artery tips but also observed in watershed regions (data not shown). Thus, several aECs upon MI upregulate Apj expression indicating change in cell fate, in response to MI.

Together, our in vivo data suggest that single aECs exiting preexisting artery tips which are the drivers of artery reassembly, show reduced Cx40 expression, increased Apj expression, and proliferate during the course of neonatal artery reassembly. All 3 features, that is, lack of mature cell marker, increase in early angiogenic marker, and cell cycle reentry are indicative of dedifferentiation. Thus, we propose that preexisting aECs dedifferentiate and proliferate to build coronary collateral arteries during artery reassembly (Figure 4H).

### Adult aECs Do Not Dedifferentiate, Upon MI

**Figure 5.**
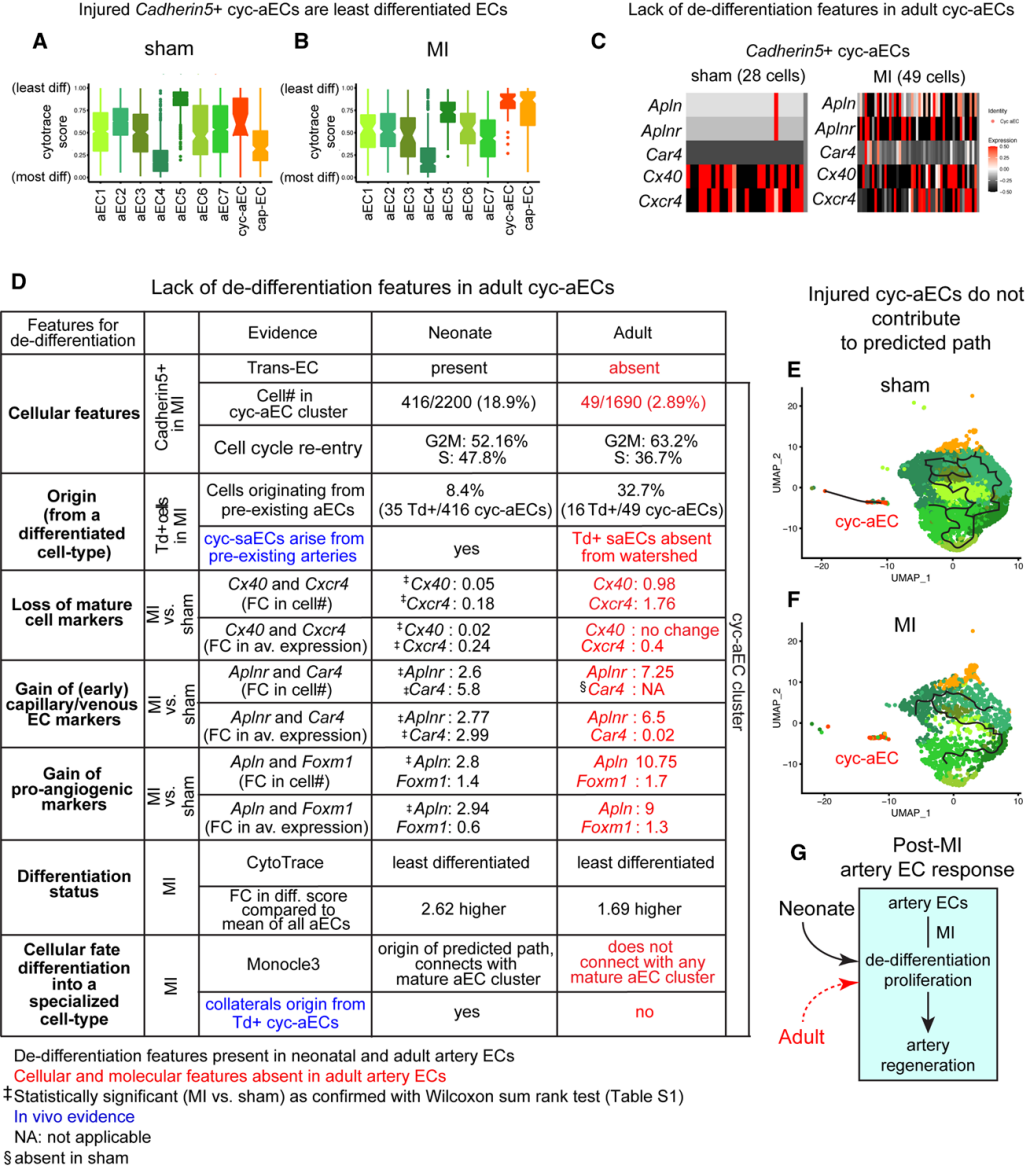
**Analyses of dedifferentiation features in adult myocardial infarction (MI) cells. A** and **B**, Box plots illustrating a relative differentiation state of adult *Cadherin5*^+^ (**A**) sham and (**B**) MI cells, with scores obtained from Cellular Trajectory Reconstruction Analysis using Gene counts and Expression. **C**, Heatmap of adult sham and MI cells showing the expression of *Apln*, *Aplnr*, *Car4*, *Cx40*, and *Cxcr4*, in their respective cyc–artery endothelial cell (cyc-aEC) cluster. **D**, Comparison of dedifferentiation features in *Cadherin5*^+^ neonatal and adult cyc-aEC cluster. **E** and **F**, Pseudo time analysis of adult *Cadherin5*^+^ cells from (**E**) sham and (**F**) MI group. **G**, Schematic showing post-MI neonatal aECs accomplish artery regeneration by undergoing dedifferentiation and proliferation; a phenomenon rarely observed in adults. Dotted line, minimal contribution. # indicates number; av., average; cap-EC, capillary EC; diff, differentiation; FC, fold change; Td, TdTomato; Trans-EC, transitioning EC; and UMAP, Uniform Manifold Approximation and Projection.

We next checked if adult MI *Cadherin5*^+^ cycling aECs (Figure 2I) demonstrate any characteristics indicative of dedifferentiation. Although adult MI cycling aEC cluster was the least differentiated aEC cluster (Figure 5A and 5B), its CytoTRACE score was comparable to a mature artery cluster—aEC5 in MI (Figure 5B). Expression of proangiogenic marker *Apln*, and its receptor *Aplnr* were seemingly upregulated in this cluster, but there was no change in expression of *Car4*, *Cx40*, or *Cxcr4* (Figure 5C). We analyzed the cellular and molecular features of neonatal cyc-aECs, which were indicative of dedifferentiation, and checked those indicators in adult cyc-aECs (Figure 5D). Some of the key dedifferentiation features absent in adult cyc-aECs were—(1) their adequate number, which is representative of their ability to self-renew/ reenter cell cycle, (2) loss of mature cell markers, and (3) gain of microvascular/ proangiogenic markers (shown in red, Figure 5D). For instance, in contrast to neonatal MI cyc-aECs, adult MI cyc-aECs, showed almost no change in the number of *Cx40*-expressing cells (Figure 5D). Adult cells expressing *Cxcr4* in this group, increased by 1.76-fold (Figure 5D). The number of cells expressing *Apln*, *Aplnr*, and *Car4* in the adult cyc-aECs was increased upon MI. However, these changes in cell numbers or gene expression levels, were statistically insignificant when compared between adult sham and MI groups, using Wilcoxon sum-rank test (Figure 5D; Table S1). Furthermore, cycling aEC cluster did not contribute to the predicted path associated with mature aECs (aEC1–7), even in MI group (Figure 5E and 5F). We confirmed the same with RNA velocity analyses (Figure S5H). Thus, unlike neonate, adult cycling aECs were neither connected to aECs in trajectory analyses (Figure 5E and 5F) nor were lineage traced from preexisting arteries (Figure 2J and 2K). Together, adult cycling aEC population fails to drive artery regeneration (Figure 5G) due to its limited number and inability to dedifferentiate (Figure 5D).

### Arterial VegfR2 Promotes aEC Proliferation During Artery Reassembly

**Figure 6.**
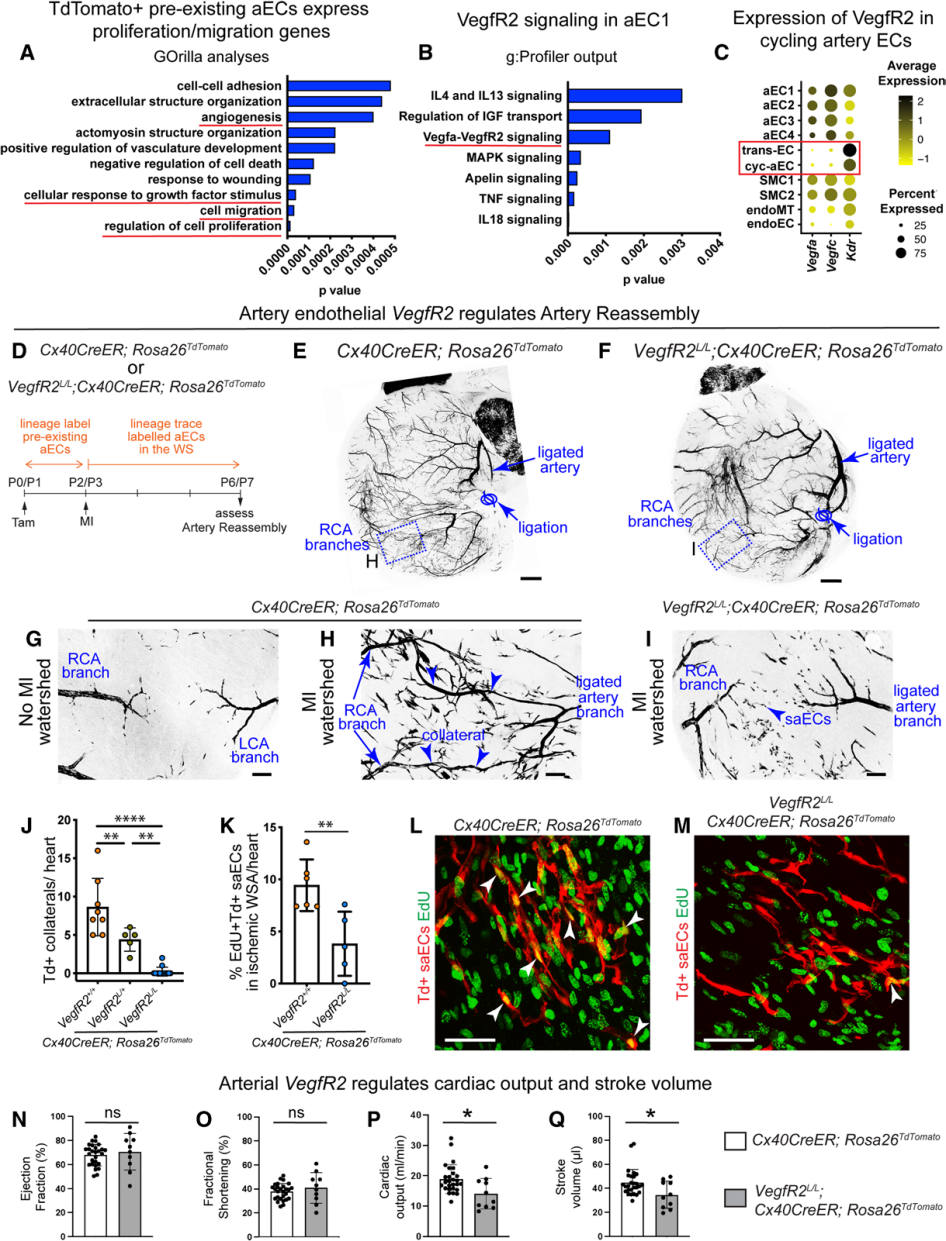
**Vegf (vascular endothelial growth factor) pathway in neonatal artery reassembly and cardiac function. A**, Results from Gene Ontology analysis on TdTomato^+^ cells from neonatal myocardial infarction (MI) group (with sham as background), using the tool Gene Ontology Enrichment Analysis and Visualization Tool (GOrilla). *P* values for regulation of cell proliferation are 0.000000000331 and cell migration is 0.00000712. **B**, Pathway analysis on genes expressed in neonatal artery endothelial cell 1 (aEC1) cluster, using g:Profiler. **C**, DotPlot showing average expression and percentage of cells expressing *Vegfa*, *Vegfc*, and *Kdr* (Vegf receptor-2 [*VegfR2*]) in all *Cadherin5*^+^ cells. **D**, Experimental setup to test the role of arterial VegfR2 in neonatal artery reassembly. **E** and **F**, Representative image of (**E**) control and (**F**) *VegfR2* aEC-specific knockout whole hearts, 4 days post-MI. **G** through **I**, Representative confocal images of postnatal day (P) 6 watershed regions from (**G**) uninjured, (**H**) MI control, and (**I**) MI *VegfR2* knockout hearts. MIs were performed at P2. Lineage-traced aECs are shown in black. **J**, Quantification of *Cx40CreER* lineage–traced collateral artery numbers observed 4 days post-MI. Control vs heterozygous (*P*=0.0058), heterozygous vs knockout (*P*=0.0029), and control vs knockout (*P*<0.0001). **K**, Quantification of percentage of 5-ethynyl-2′-deoxyuridine (EdU)^+^ Tdtomato^+^ single aECs (saECs) in watershed regions from *Cx40CreER* lineage–traced hearts, 4 days post-MI. **P=0.0083. **L** and **M**, Representative confocal images of EdU^+^ Tdtomato^+^ saECs (white arrowheads) in watershed regions from *Cx40CreER* lineage–traced hearts, (**L**) with or (**M**) without arterial *VegfR2*, 4 days post-MI. **N** through **Q**, Graphs showing various parameters of cardiac function as measured in adult wild-type (n=27) and *VegfR2* aEC-specific knockout mice (n=10). *P*=0.5067 (ejection fraction), *P*=0.3401 (Fractional Shortening), *P*=0.0108 (cardiac output), *P*=0.0186 (stroke volume). Scale bars: **E** and **F**, 500 µm; **G–I**, 100 µm; **L** and **M**, 50 µm. Cyc-EC indicates cycling EC; endo-EC, endocardial EC; endo-MT, endothelial to mesenchymal transition; IL, interleukin; LCA, left coronary artery; MAPK, mitogen-activated protein kinase; RCA, right coronary artery; SMC, smooth muscle cell; TNF, tumor necrosis factor; Trans-EC, transitioning EC; and WSA, watershed area.

Preexisting aECs drive collateral artery formation in neonatal mice, only upon MI.^17,62^ We isolated all genes from *Cx40CreER* lineage–traced TdTomato^+^ injured aECs (Figure 1F) and performed gene ontology enrichment analysis and visualization (GOrilla). This allowed us to identify biological processes triggered upon MI. Key vascular responses, such as angiogenesis, migration, and proliferation, were significantly upregulated upon MI (Figure 6A). Artery reassembly is a systematic step-wise cellular event where aEC migration and proliferation are crucial. Thus, our neonatal single-cell data set contains the aEC population, including those that drive artery reassembly, and subsequent neonatal cardiac regeneration.

aEC1, in neonatal MI data set, is the only aEC cluster associated with the predicted trajectory and connected with cycling aECs (Figure 3F). We performed pathway analyses on *Cadherin5*^+^ aEC1 cells using g:Profiler and filtered pathways from various databases, based on their statistical significance (*P*>0.05). Many pathways relevant to vascular development and injury response were enriched in aEC1 such as apelin, TNF, and Interleukin signaling^63^ (Figure 6B). Vegfa-VegfR2 signaling (*P*=0.0011) was of particular interest (Figure 6B), as it has established molecular functions in mouse coronary artery development^48,64^ and neovascularization upon injury in different tissues.^65,66^

We checked the expression levels of ligands (*Vegfa*, *Vegfc*) and a major receptor (*VegfR2* or *Kdr*) associated with coronary morphogenesis, in our data set. While *Vegfa* and *Vegfc* were expressed in aECs (aEC1–4), *VegfR2* was specifically enriched in cycling aEC and trans-EC population (Figure 6C). The *VegfR2* expression, was significantly different in cyc-aECs, when compared between neonatal sham and MI groups, using Wilcoxon sum-rank test (Table S1). When immunostained for VegfR2, the protein was located in coronary aECs of small arteries, artery tips, and capillary ECs (Figure S8A). The levels of VegfR2 protein in arteries were lower as compared to capillaries, and the protein was almost always absent in large arteries (Figure S8A). This could mean an overall reduced expression of arterial VegfR2 (as compared to capillaries) or expression of VegfR2 by a smaller subset of arterial ECs. Both EC types (arteries and capillaries) facilitate artery reassembly, either by directly participating in the process (aECs) or providing the Cxcl12 ligand (cap-ECs).^17^ Given VegfR2 expression in both, capillaries and small arteries, we hypothesized that Vegf pathway has a role in artery reassembly, post-MI.

We analyzed the role of arterial *VegfR2* in artery reassembly, in vivo. Control (*Cx40CreER*; *Rosa26^TdTomato^*) and aEC-specific, conditional *VegfR2* knockout (*VegfR2^L/L^*; *Cx40CreER*; *Rosa26^TdTomato^*) neonates received tamoxifen at P0/P1 which allowed labeling of preexisting aECs. These hearts were subjected to MI at P2/P3 and hearts were harvested 4 days post-MI, at P6/P7 (Figure 6D). Whole heart imaging allowed us to visualize the entire arterial network of the heart (Figure 6E and 6F) as well as focus into regions of interest such as the watershed region where new collaterals are expected to appear after injury (Figure 6G through 6I). Control and *VegfR2* artery-specific knockout hearts did not illustrate any gross differences in their baseline artery architecture, without MI (Figure S8B through S8D). Watershed regions from sham (data not shown) or no injury (Figure 6G) hearts, did not show any collateral arteries lineage labeled with TdTomato, suggesting absence of artery reassembly in nonischemic hearts. Upon MI, control hearts consistently showed several (average of 8) *Cx40CreER* lineage–traced (TdTomato^+^) collateral arteries as observed earlier^17^ (Figure 6E and 6H). In contrast, we rarely observed any such collateral arteries in *VegfR2* artery-specific knockout hearts (Figure 6F, 6I, and 6J). This suggests an artery-specific role of VegfR2 in executing artery reassembly, upon MI.

We next investigated the cause for lack of TdTomato^+^ collateral arteries in *VegfR2* knockout watershed regions. Artery reassembly is a 3-step cellular event—migration, proliferation, and coalescence, of aECs. We first assessed the ability of TdTomato^+^ cells to migrate into the watershed. Qualitative and quantitative observations suggested that the number of *Cx40CreER* lineage–traced single aECs, observed in the watershed regions, were comparable in control and knockout MI hearts (Figure 6H and 6I; Figure S8E through S8I). This suggests that in absence of arterial VegfR2, the migration of aECs from their resident arteries into the watershed region is less likely affected. We next investigated the proliferation step and injected neonates with EdU, 1 hour before harvesting the hearts. We then quantified the number of proliferating single aECs in the MI watershed regions. Interestingly, *VegfR2* knockout watershed regions showed a ≈2.4-fold reduction in the number of proliferating single aECs as compared to control hearts (Figure 6K through 6M; Figure S9). Such reduction in number of proliferating single aECs was also observed in nonregenerative P7 injured hearts (Figure 2O). Thus, arterial VegfR2 regulates neonatal aEC proliferation, post-MI. A defect in VegfR2-mediated artery cell proliferation could also affect subsequent coalescence of TdTomato^+^ cells and successful execution of artery reassembly, as observed in *VegfR2* knockout hearts (Figure 6J).

Functional coronary collateral arteries built by artery reassembly promote cardiac regeneration. Since arterial deletion of *VegfR2* reduced number of collateral arteries, we tested if this neonatal *VegfR2* deletion affects cardiac function in adults. We performed MIs at neonatal stage (P2) and checked several parameters for cardiac function 30 days post-MI. Although parameters like ejection fraction and fractional shortening did not change significantly (Figure 6N and 6O), cardiac output and stroke volume were significantly reduced (Figure 6P and 6Q) in *VegfR2* arterial EC-specific knockouts. Thus, arterial VegfR2 facilitates neonatal artery reassembly that regulates cardiac function in adult mice.

We next investigated if Vegf ligands and receptors are expressed in adult ECs. The expression of Vegf ligands, *Vegfa* and *Vegfc*, were mostly observed in mature aEC clusters (Figure S10A) like in neonatal data (Figure 6C). The receptor *VegfR2* (*Kdr*), was upregulated in capillary ECs, as expected (Figure S10A). Interestingly, at least at the mRNA level, *VegfR2* was downregulated in ≈75% of actively cycling aECs (Figure S10A). This is in contrast to the upregulated expression of *VegfR2* in neonatal cyc-aECs (Figure 6C). When compared with sham group, *VegfR2* was significantly downregulated in adult MI cyc-aEC cluster (Table S1). Given the functional significance of arterial *VegfR2* in neonatal aEC proliferation (Figure 6K through 6M), the lack of *VegfR2* expression in residual adult cycling aECs, could explain depleted potential for aEC proliferation in injured adults. In summary, our data indicate that arterial VegfR2 drives, specifically, the proliferation step of artery reassembly in injured neonatal mice (Figure S10B).

## DISCUSSION

In this study, we utilize both in silico analyses and in vivo experiments to explain the age-dependent aEC responses to cardiac injury. First, with single-cell sequencing data analyses, we show that coronary aECs, an otherwise stably differentiated cardiac cell type, undergo injury-induced dedifferentiation in neonates, but not in adults. In this study, we defined aEC dedifferentiation as a biological process involving 4 major cellular/molecular features (1) downregulation of mature aEC cell markers, (2) expression of early and angiogenic markers, (3) cell cycle reentry, and (4) subsequent differentiation into more aECs to build new collateral arteries by artery reassembly. With the combination of bioinformatics and mouse experiments, we show that the ability of aECs to dedifferentiate correlates with their ability to proliferate. Both cellular responses—artery cell dedifferentiation and proliferation—are specific to neonatal regenerative window. Second, using in vivo experiments, we discover a second artery-specific receptor (apart from Cxcr4, which controls artery cell migration during artery reassembly^17^), which regulates proliferation of aECs in response to neonatal MI. Expression of this receptor, *VegfR2*, is downregulated in adult cycling aECs and could explain absence of artery reassembly in adults. Finally, our data suggest that each step of artery reassembly is regulated by a distinct signaling axis, and the successful execution of each step will require activation of these molecular pathways in the right space and time. In summary, we highlight the differences in age-dependent molecular profiles of mouse coronaries and propose a mechanism for reduced plasticity of aECs and lack of artery reassembly in nonregenerative P7 or adult hearts.

We used single-cell RNA sequencing to investigate MI-induced aEC response in neonates and adults. We obtained fewer aECs upon MI as compared to sham. This was observed in both neonates and adults. In our data set, the time points when the neonatal or adult hearts were harvested represent a state when cardiac regeneration has not been completed yet. At the chosen time points, while many vascular regenerative processes have been initiated, it is likely that cell death would still be in progression. This phenomenon is also reflected in *Cx40CreER* lineage–traced TdTomato aECs. Such small fraction of aECs observed in our data set have also been reported in other neonatal and adult data sets^20,31,32^ and likely represent extensive cell death following coronary ligation and a vascular environment before complete cardiac recovery.

TdTomato cells in our MI data sets were ≈17-fold less in neonates and ≈5-fold less in adults as compared to their sham counterparts. We hence analyzed all ECs which included both TdTomato^+^ and TdTomato^−^ cells. The analyses of *Cadherin5*+ all ECs allowed us to build several hypotheses which were tested in vivo. Using genetic lineage tracing and confocal imaging of whole hearts, we show that post-MI, (1) neonatal aECs also originate from sources other than preexisting aECs, (2) during artery reassembly cycling preexisting aECs downregulate mature aEC marker (*Cx40*) and upregulate marker for capillary/vein (*Apj*) from which aECs originate during development, (3) aEC proliferation is minimal in nonregenerative P7 hearts when subjected to MI and finally, (4) injury-induced aEC proliferation is regulated by VegfR2-mediated pathway, which subsequently impacts cardiac function of adult mice. These in vivo results were based on the findings from our single-cell data set. Thus, despite their low numbers in our single-cell data sets, the aEC population is representative of postinjury vascular niche in vivo.

### Dedifferentiation and Proliferation Are Coupled During Cardiac Regeneration

New cardiomyocytes originate from preexisting cardiomyocytes upon injury^15,16^ by dedifferentiating and reentering cell cycle.^67,68^ Cardiomyocyte dedifferentiation has been elucidated upon adult zebrafish cardiac injury via disassembly of sarcomeric structures, detachment of cardiomyocytes from one another, and expression of early embryonic genes (αSMA, embryonic myosin heavy chain, and Gata4).^67–69^ Post-MI adult cardiomyocytes also demonstrate somewhat limited dedifferentiation potential indicated by downregulation of mature cardiomyocyte functions such as hypertrophy, contractility, electrical conduction, and upregulation of several early genes like *αSMA*, *Gata4*, *Runx1*, and *Dab2*.^70^ Unlike cardiomyocytes, EC dedifferentiation has not been reported in any cardiac injury model. We show here, for the first time, that neonatal mouse coronary aECs undergo dedifferentiation upon MI; a process limited in adults.

Since there are no objective indicators of aEC pluripotency, one can argue that changes in gene expression or phenotypes could be a reflection of their plasticity and not necessarily reversion of cell fate into a dedifferentiated state. It is also possible that the number of dedifferentiated cells observed in cyc-aEC and trans-EC clusters of uninjured hearts simply increases upon MI. To address this, we combine single-cell analyses and genetic lineage tracing. Lineage-labeled noncycling aECs in MI hearts can be sequentially traced into cycling single aECs in the watershed, and consequently into noncycling aECs on the coronary collaterals. We analyzed the transiently cycling aECs and show that several indicators for dedifferentiation were enhanced in neonates. One such indicator was reduced expression of *Cx40* in cyc-aECs upon MI. This expression changed minimally in sham cyc-aEC group when compared with sham mature aECs. This could be an indication of their low cycling potential due to lack of appropriate ischemia-induced molecular signals which drive dedifferentiation and artery reassembly. A second indicator was presence of Apj lineage in Cx40^+^ aECs. It is possible that Apj lineage–traced capillaries differentiate into aECs, a phenomenon called arterialization already shown to contribute (though minimally) to collateral formation by artery reassembly.^17^ Our in vivo data cannot distinguish between differentiation of capillary ECs into aECs and upregulation of capillary marker (Apj) in arteries. However, the unique location and morphology of cells allowed us to confidently identify Cx40^+^ single aECs and conclude that at least a subpopulation of aECs, upon MI, express Apj—an indicative of vascular dedifferentiation.

The dedifferentiation indicators were absent in adults. This finding positively correlated with the ability of aECs to reenter cell cycle. Neonates had much higher number of cycling aECs than P7 or adults. This finding was verified via 2 independent methods—single-cell gene expression analyses of cyc-aECs and presence of *Cx40CreER*^+^ EdU^+^ cells in an in vivo cardiac MI model. Thus, lack of dedifferentiation limits the number of cycling aECs in nonregenerative hearts and consequently affects their ability to form collateral arteries by artery reassembly.

### Reactivation of Developmental Pathways Upon Injury?

Several molecular pathways relevant to vascular development regulate EC response to injury.^71^ For instance, Notch,^72^ Apelin,^55^ Cxcl12^73–76^ signaling, in mice, regulate different aspects of vascular development and remodeling during cardiac morphogenesis; ranging from EC fate determination to regulation of different EC responses such as migration, proliferation, and tube formation. Interestingly, some of these pathways are also associated with EC regeneration upon injury,^17,58,77^ making them potential therapeutic candidates for treating vascular conditions in human patients.^78^ Further studies are required, and will elucidate if (and how) the cellular and molecular mechanisms associated with these signaling pathways, during development and injury responses, are similar.

Vegf signaling drives coronary development in zebrafish and mouse hearts. In mice, Vegf signaling drives migration of coronary endothelial^53,79^ and endo-ECs^64^ in developing hearts, and proliferation of coronary ECs in heart explant cultures.^80^ In zebrafish, *vegfa* mutants show reduced EC proliferation possibly via PI3K signaling.^81^ Upon cardiac injury, endocardial Vegfa signaling^77^ and Vegfc-mediated (Cxcl8a/Cxcr1–driven) coronary EC proliferation^82^ execute revascularization and cardiac regeneration of ischemic hearts. In this study, we highlight the role of VegfR2-mediated signaling in aEC response triggered by neonatal cardiac injury. We show that VegfR2 in neonates is expressed in coronary aECs, deletion of which, significantly reduced the number of collateral arteries formed via artery reassembly.

Our data show that artery tips do not proliferate. Proliferation is rather demonstrated by single aECs which originate from these artery tips. This suggests that migration of preexisting aECs could precede aEC proliferation in the watershed. Appearance of *VegfR2* depleted single aECs in ischemic watershed regions suggested that aEC migration was not affected. In fact, the TdTomato+ cells were increased in the *VegfR2*-depleted hearts as compared to wild-type hearts. This could be because TdTomato+ single aECs accumulated in the watershed due to inability to coalesce and form collateral arteries. VegfR2-depleted single aECs showed reduced proliferation potential, which could account for lack of lineage-traced collateral arteries in the knockout hearts. Proliferation of aECs is followed by their coalescence into collateral arteries. Thus, we cannot exclude the possible role of Vegf in coalescence of artery cells, or the vascular steps that follow, like lumenization or maturation of collateral arteries. It is also possible that because of reduced rate of arterial coalescence, *VegfR2* null saECs reduce their proliferation rate as a feedback mechanism. This is also evident from higher number of *VegfR2* null saECs present in post-MI watershed.

The precise source of Vegf ligand in injured mouse hearts could be the endocardium,^83^ myocardium (Vegfa),^64,84^ epicardium (Vegfc^53^ or Vegfa^85^), or cardiac ECs,^82^ thus suggesting an autocrine or paracrine signaling as observed in injured zebrafish hearts.^82^ In our neonatal mouse single-cell data set, we found both *Vegfa* and *Vegfc* being expressed by mature aECs (aEC1–4), but at lower levels by cyc-aECs (or by trans-ECs, SMCs, endo-MT ECs, endo ECs), indicating a possible cell autonomous mechanism. However, an alternate source of Vegf ligand, such as cardiomyocytes, fibroblasts, or immune cells, which are excluded from our data set, cannot be neglected.

### Role of Vegf Pathway in Human Heart Disease and Its Treatment

Single nucleotide polymorphisms in both introns and exons of *KDR* (murine *VegfR2*) are strongly associated with coronary heart disease (CHD).^86^ These single nucleotide polymorphisms can either modulate the affinity to ligand or alter E2F binding to *KDR* promoter, subsequently, affecting *KDR* activity and downstream signaling.^86^ Elevated levels of circulating VEGF ligands are positively and significantly associated with CHD,^87^ coronary artery disease,^88,89^ and coronary artery lesions.^90,91^ Several single nucleotide polymorphisms affect VEGF levels^87,89^ and may account for the variation observed in revascularization abilities in individuals across different genetic backgrounds. At least 3 such single nucleotide polymorphisms in *VEGFA* are strongly associated with increased risk of CHD/MI^92^ and one with coronary collateral vessel perfusion.^93^ How the association of VEGF/KDR signaling axis with CHD may serve for better prognosis or indicate the need for vascularization, remains unclear.

Preclinical studies in animals with sparse^94^ to no^49^ preexisting collateral arteries have consistently elucidated role of Vegf in ischemia-induced collateral formation. On the contrary, numerous past and ongoing clinical trials with *VEGF* alone, or combined with proangiogenic/regenerative factors, have been concluded with little to no success (data available at clinicaltrials.gov).^94,95^ The delivery methods appear to be safe, but the angiogenic effects, particularly the efficacy of *VEGF* gene therapies in stimulating reperfusion via collateral arteries are minimal.^94^ Mechanistic details of Vegf’s mode of action, in orchestrating vascular cell responses that lead to collateral formation, could aid designing clinical trials with higher efficacies.

We show that mouse coronary ECs in MI hearts dedifferentiate and then expand in VegfR2-dependent manner. The mere presence of a significant number of single aECs in a limited watershed area could be sufficient to create new arteries.^39^ Thus, it will be interesting to see if this threshold density of aECs is accomplished by Vegf-mediated proliferation. Similarly, identifying the molecular drivers of EC dedifferentiation is also crucial, and when combined with Vegf, could promote collaterals in individuals with CHD and coronary artery disease.

## ARTICLE INFORMATION

### Acknowledgments

The authors thank the following facilities for their technical support: Stanford Genome Sequencing Center, Stanford shared fluorescence-activated cell sorting (FACS) facility, Animal Care and Resource Center at National Centre for Biological Sciences, Mouse Genome Engineering Facility at National Centre for Biological Sciences, Central Imaging and Flow Cytometry Facility at National Centre for Biological Sciences, Dr Dhandapany Perundurai’s lab from Institute For Stem Cell Science and Regenerative Medicine (InStem), for their technical help with echocardiography. S. Das and K. Red-Horse conceived the idea. S. Das, K. Red-Horse, Y.J. Woo provided resources. G. Arolkar performed experiments with neonatal myocardial infarction (MI) model. S.K. Kumar and S. Kumar performed bioinformatics analyses. S. Das, G. Arolkar, and S.K. Kumar wrote the article. H. Wang performed adult MI surgeries. P.E. Rios Coronado and B. Bishnoi performed some neonatal MI surgeries. S. Das and K.M. Gonzalez performed FACS.

### Sources of Funding

This work is supported by National Centre for Biological Sciences/Tata Institute of Fundamental Research core funding and Department of Biotechnology (DBT)/Wellcome Trust India Alliance Intermediate Fellowship to S. Das (IA/I/20/2/505205). Y.J. Woo is supported by National Institutes of Health (5R01HL089315-11). K. Red-Horse is supported by National Institutes of Health (R01-HL128503) and is a Howard Hughes Medical Investigator (HHMI). H. Wang is supported by American Heart Association (18POST33990223). K.M. Gonzalez is supported by Gabilan Stanford Graduate Fellowship, National Science Foundation–Graduate Research Fellowship Program (NSF-GRFP). S. Kumar is supported by DBT/Wellcome Trust India Alliance Intermediate Fellowship to S. Das. P.E. Rios Coronado is supported by the National Institute of General Medical Sciences (NIGMS) of the National Institutes of Health (NIH T32GM007276) and NSF-GRFP (DGE-1656518).

### Disclosures

None.

### Supplemental Material

Figure S1-10 and legends

Table S1

Major Resources Table

Graphical abstract

## Supplementary Material

## References

[1] Seiler C Stoller M Pitt B Meier P. The human coronary collateral circulation: development and clinical importance. Eur Heart J. 2013;34:2674–2682. doi: 10.1093/eurheartj/eht195 23739241 10.1093/eurheartj/eht195

[2] Sabia PJ Powers ER Ragosta M Sarembock IJ Burwell LR Kaul S. An association between collateral blood flow and myocardial viability in patients with recent myocardial infarction. N Engl J Med. 1992;327:1825–1831. doi: 10.1056/nejm199212243272601 1448120 10.1056/NEJM199212243272601

[3] Meier P Gloekler S Zbinden R Beckh S de Marchi SF Zbinden S Wustmann K Billinger M Vogel R Cook S. Beneficial effect of recruitable collaterals: a 10-year follow-up study in patients with stable coronary artery disease undergoing quantitative collateral measurements. Circulation. 2007;116:975–983. doi: 10.1161/CIRCULATIONAHA.107.703959 17679611 10.1161/CIRCULATIONAHA.107.703959

[4] Habib GB Heibig J Forman SA Brown BG Roberts R Terrin ML Bolli R. Influence of coronary collateral vessels on myocardial infarct size in humans. Results of phase I thrombolysis in myocardial infarction (TIMI) trial. The TIMI investigators. Circulation. 1991;83:739–746. doi: 10.1161/01.cir.83.3.739 1900223 10.1161/01.cir.83.3.739

[5] Clayton JA Zhang H Pomp D Faber JE Chalothorn D. Collateral density, remodeling, and VEGF-A expression differ widely between mouse strains. Physiol Genomics. 2007;30:179–191. doi: 10.1152/physiolgenomics.00047.2007 17426116 10.1152/physiolgenomics.00047.2007

[6] Teunissen PFA Horrevoets AJG van Royen N. The coronary collateral circulation: genetic and environmental determinants in experimental models and humans. J Mol Cell Cardiol. 2012;52:897–904. doi: 10.1016/j.yjmcc.2011.09.010 21959171 10.1016/j.yjmcc.2011.09.010

[7] Maxwell MP Hearse DJ Yellon DM. Species variation in the coronary collateral circulation during regional myocardial ischaemia: a critical determinant of the rate of evolution and extent of myocardial infarction. Cardiovasc Res. 1987;21:737–746. doi: 10.1093/cvr/21.10.737 3440266 10.1093/cvr/21.10.737

[8] Chalothorn D Faber JE. Formation and maturation of the native cerebral collateral circulation. J Mol Cell Cardiol. 2010;49:251–259. doi: 10.1016/j.yjmcc.2010.03.014 20346953 10.1016/j.yjmcc.2010.03.014 PMC2885464

[9] Perovic T Harms C Gerhardt H. Formation and maintenance of the natural bypass vessels of the brain. Front Cardiovasc Med. 2022;9:1–6. doi: 10.3389/fcvm.2022.778773 10.3389/fcvm.2022.778773 PMC8980479 35391845

[10] Gabhann F Peirce SM. Collateral capillary arterialization following arteriolar ligation in murine skeletal muscle. Microcirculation. 2010;17:333–347. doi: 10.1111/j.1549-8719.2010.00034.x 20618691 10.1111/j.1549-8719.2010.00034.x PMC2907254

[11] Scholz D Ziegelhoeffer T Helisch A Wagner S Friedrich C Podzuweit T Schaper W. Contribution of arteriogenesis and angiogenesis to postocclusive hindlimb perfusion in mice. J Mol Cell Cardiol. 2002;34:775–787. doi: 10.1006/jmcc.2002.2013 12099717 10.1006/jmcc.2002.2013

[12] Zhang H Prabhakar P Sealock R Faber JE. Wide genetic variation in the native pial collateral circulation is a major determinant of variation in severity of stroke. J Cereb Blood Flow Metab. 2010;30:923–934. doi: 10.1038/jcbfm.2010.10 20125182 10.1038/jcbfm.2010.10 PMC2949178

[13] Chalothorn D Faber JE. Strain-dependent variation in collateral circulatory function in mouse hindlimb. Physiol Genomics. 2010;42:469–479. doi: 10.1152/physiolgenomics.00070.2010 20551146 10.1152/physiolgenomics.00070.2010 PMC2929883

[14] Clayton JA Chalothorn D Faber JE. Vascular endothelial growth factor-a specifies formation of native collaterals and regulates collateral growth in ischemia. Circ Res. 2008;103:1027–1036. doi: 10.1161/CIRCRESAHA.108.181115 18802023 10.1161/CIRCRESAHA.108.181115 PMC2729271

[15] Porrello ER Mahmoud AI Simpson E Hill JA Richardson JA Olson EN Sadek HA. Transient regenerative potential of the neonatal mouse heart. Science. 2011;331:1078–1080. doi: 10.1126/science.1200708 21350179 10.1126/science.1200708 PMC3099478

[16] Porrello ER Mahmoud AI Simpson E Johnson BA Grinsfelder D Canseco D Mammen PP Rothermel BA Olson EN Sadek HA. Regulation of neonatal and adult mammalian heart regeneration by the miR-15 family. Proc Natl Acad Sci USA. 2013;110:187–192. doi: 10.1073/pnas.1208863110 23248315 10.1073/pnas.1208863110 PMC3538265

[17] Das S Goldstone AB Wang H Farry J D'Amato G Paulsen MJ Eskandari A Hironaka CE Phansalkar R Sharma B. A unique collateral artery development program promotes neonatal heart regeneration. Cell. 2019;176:1128–1142.e18. doi: 10.1016/j.cell.2018.12.023 30686582 10.1016/j.cell.2018.12.023 PMC6435282

[18] Aurora AB Porrello ER Tan W Mahmoud AI Hill JA Bassel-Duby R Sadek HA Olson EN. Macrophages are required for neonatal heart regeneration. J Clin Invest. 2014;124:1382–1392. doi: 10.1172/JCI72181 24569380 10.1172/JCI72181 PMC3938260

[19] Wang Y Yao F Wang L Li Z Ren Z Li D Zhang M Han L Wang SQ Zhou B. Single-cell analysis of murine fibroblasts identifies neonatal to adult switching that regulates cardiomyocyte maturation. Nat Commun. 2020;11:2585. doi: 10.1038/s41467-020-16204-w 32444791 10.1038/s41467-020-16204-w PMC7244751

[20] Wang Z Cui M Shah AM Tan W Liu N Bassel-Duby R Olson EN. Cell-type-specific gene regulatory networks underlying murine neonatal heart regeneration at single-cell resolution. Cell Rep. 2020;33:108472. doi: 10.1016/j.celrep.2020.108472 33296652 10.1016/j.celrep.2020.108472 PMC7774872

[21] Hao Y Hao S Andersen-Nissen E Mauck WM Zheng S Butler A Lee MJ Wilk AJ Darby C Zager M. Integrated analysis of multimodal single-cell data. Cell. 2021;184:3573–3587.e29. doi: 10.1016/j.cell.2021.04.048 34062119 10.1016/j.cell.2021.04.048 PMC8238499

[22] Cao J Spielmann M Qiu X Huang X Ibrahim DM Hill AJ Zhang F Mundlos S Christiansen L Steemers FJ. The single-cell transcriptional landscape of mammalian organogenesis. Nature. 2019;566:496–502. doi: 10.1038/s41586-019-0969-x 30787437 10.1038/s41586-019-0969-x PMC6434952

[23] Melsted P Booeshaghi AS Liu L Gao F Lu L Min KHJ da Veiga Beltrame E Hjörleifsson KE Gehring J Pachter L. Modular, efficient and constant-memory single-cell RNA-seq preprocessing. Nat Biotechnol. 2021;39:813–818. doi: 10.1038/s41587-021-00870-2 33795888 10.1038/s41587-021-00870-2

[24] la Manno G Soldatov R Zeisel A Braun E Hochgerner H Petukhov V Lidschreiber K Kastriti ME Lönnerberg P Furlan A. RNA velocity of single cells. Nature. 2018;560:494–498. doi: 10.1038/s41586-018-0414-6 30089906 10.1038/s41586-018-0414-6 PMC6130801

[25] Gulati GS Sikandar SS Wesche DJ Manjunath A Bharadwaj A Berger MJ Ilagan F Kuo AH Hsieh RW Cai S. Single-cell transcriptional diversity is a hallmark of developmental potential. Science. 2020;367:405–411. doi: 10.1126/science.aax0249 31974247 10.1126/science.aax0249 PMC7694873

[26] Eden E Navon R Steinfeld I Lipson D Yakhini Z. GOrilla: a tool for discovery and visualization of enriched GO terms in ranked gene lists. BMC Bioinf. 2009;10:48. doi: 10.1186/1471-2105-10-48 10.1186/1471-2105-10-48 PMC2644678 19192299

[27] Raudvere U Kolberg L Kuzmin I Arak T Adler P Peterson H Vilo J. G:Profiler: a web server for functional enrichment analysis and conversions of gene lists (2019 update). Nucleic Acids Res. 2019;47:W191–W198. doi: 10.1093/nar/gkz369 31066453 10.1093/nar/gkz369 PMC6602461

[28] Miquerol L Thireau J Bideaux P Sturny R Richard S Kelly RG. Endothelial plasticity drives arterial remodeling within the endocardium after myocardial infarction. Circ Res. 2015;116:1765–1771. doi: 10.1161/CIRCRESAHA.116.306476 25834185 10.1161/CIRCRESAHA.116.306476

[29] Miquerol L Meysen S Mangoni M Bois P van Rijen HVM Abran P Jongsma H Nargeot J Gros D. Architectural and functional asymmetry of the His–Purkinje system of the murine heart. Cardiovasc Res. 2004;63:77–86. doi: 10.1016/j.cardiores.2004.03.007 15194464 10.1016/j.cardiores.2004.03.007

[30] Mahmoud A Porrello E Kimura W. Surgical models for cardiac regeneration in neonatal mice. Nat Protoc. 2014;9:305–311. doi: 10.1038/nprot.2014.021.Surgical 24434799 10.1038/nprot.2014.021 PMC3977725

[31] Farbehi N Patrick R Dorison A Xaymardan M Janbandhu V Wystub-Lis K Ho JW Nordon RE Harvey RP. Single-cell expression profiling reveals dynamic flux of cardiac stromal, vascular and immune cells in health and injury. Elife. 2019;8:e43882. doi: 10.7554/eLife.43882 30912746 10.7554/eLife.43882 PMC6459677

[32] Zhuang L Lu L Zhang R Chen K Yan X. Comprehensive integration of single-cell transcriptional profiling reveals the heterogeneities of non-cardiomyocytes in healthy and ischemic hearts. Front Cardiovasc Med. 2020;7:615161. doi: 10.3389/fcvm.2020.615161 33365332 10.3389/fcvm.2020.615161 PMC7750309

[33] Faggin E Puato M Zardo L Franch R Millino C Sarinella F Pauletto P Sartore S Chiavegato A. Smooth muscle-specific SM22 protein is expressed in the adventitial cells of balloon-injured rabbit carotid artery. Arterioscler Thromb Vasc Biol. 1999;19:1393–1404. doi: 10.1161/01.atv.19.6.1393 10364069 10.1161/01.atv.19.6.1393

[34] Ding X An Q Zhao W Song Y Tang X Wang J Chang CC Zhao G Hsiai T Fan G. Distinct patterns of responses in endothelial cells and smooth muscle cells following vascular injury. JCI Insight. 2022;7:e153769. doi: 10.1172/jci.insight.153769 36278486 10.1172/jci.insight.153769 PMC9714785

[35] Ando H Kubin T Schaper W Schaper J. Cardiac microvascular endothelial cells express α-smooth muscle actin and show low NOS III activity. Am J Physiol Heart Circ Physiol. 1999;276:H1755–H1768. doi: 10.1152/ajpheart.1999.276.5.h1755 10.1152/ajpheart.1999.276.5.H1755 10330261

[36] Lu X Dunn J Dickinson AM Gillespie JI Baudouin SV. Smooth muscle alpha-actin expression in endothelial cells derived from CD34+ human cord blood cells. Stem Cells Dev. 2004;13:521–527. doi: 10.1089/scd.2004.13.521 15588509 10.1089/scd.2004.13.521

[37] Azuma K Ichimura K Mita T Nakayama S Jin WL Hirose T Fujitani Y Sumiyoshi K Shimada K Daida H. Presence of α-smooth muscle actin-positive endothelial cells in the luminal surface of adult aorta. Biochem Biophys Res Commun. 2009;380:620–626. doi: 10.1016/j.bbrc.2009.01.135 19285011 10.1016/j.bbrc.2009.01.135

[38] Tsuji-Tamura K Morino-Koga S Suzuki S Ogawa M. The canonical smooth muscle cell marker TAGLN is present in endothelial cells and is involved in angiogenesis. J Cell Sci. 2021;134:jcs254920. doi: 10.1242/jcs.254920 34338296 10.1242/jcs.254920

[39] Raftrey B Williams IM Rios Coronado PE Fan X Chang AH Zhao M Roth R Trimm E Racelis R D’Amato G. Dach1 extends artery networks and protects against cardiac injury. Circ Res. 2021;129:702–716. doi: 10.1161/circresaha.120.318271 34383559 10.1161/CIRCRESAHA.120.318271 PMC8448957

[40] Phansalkar R Krieger J Zhao M Kolluru SS Jones RC Quake SR Weissman I Bernstein D Winn VD D'Amato G. Coronary blood vessels from distinct origins converge to equivalent states during mouse and human development. Elife. 2021;10:1–32. doi: 10.7554/eLife.70246 10.7554/eLife.70246 PMC8673841 34910626

[41] Kalucka J de Rooij LPMH Goveia J Rohlenova K Dumas SJ Meta E Conchinha NV Taverna F Teuwen L-A Veys K. Single-cell transcriptome atlas of murine endothelial cells. Cell. 2020;180:764–779.e20. doi: 10.1016/j.cell.2020.01.015 32059779 10.1016/j.cell.2020.01.015

[42] Zhang H Pu W Li G Huang X He L Tian X Liu Q Zhang L Wu SM Sucov HM. Endocardium minimally contributes to coronary endothelium in the embryonic ventricular free walls. Circ Res. 2016;118:1880–1893. doi: 10.1161/CIRCRESAHA.116.308749 27056912 10.1161/CIRCRESAHA.116.308749

[43] Conway S Molkentin J. Periostin as a heterofunctional regulator of cardiac development and disease. Curr Genomics. 2008;9:548–555. doi: 10.2174/138920208786847917 19516962 10.2174/138920208786847917 PMC2694556

[44] Torregrosa-Carrión R Piñeiro-Sabarís R Siguero-Álvarez M Grego-Bessa J Luna-Zurita L Fernandes VS MacGrogan D Stainier DYR de la Pompa JL. Adhesion G protein-coupled receptor Gpr126/Adgrg6 is essential for placental development. Sci Adv. 2021;7:eabj5445. doi: 10.1126/sciadv.abj5445 34767447 10.1126/sciadv.abj5445 PMC8589310

[45] Yucel N Axsom J Yang Y Li L Rhoades JH Arany Z. Cardiac endothelial cells maintain open chromatin and expression of cardiomyocyte myofibrillar genes. Elife. 2020;9:e55730. doi: 10.7554/eLife.55730 33315013 10.7554/eLife.55730 PMC7758065

[46] Su T Stanley G Sinha R D'Amato G Das S Rhee S Chang AH Poduri A Raftrey B Dinh TT. Single-cell analysis of early progenitor cells that build coronary arteries. Nature. 2018;559:356–362. doi: 10.1038/s41586-018-0288-7 29973725 10.1038/s41586-018-0288-7 PMC6053322

[47] Fang JS Coon BG Gillis N Chen Z Qiu J Chittenden TW Burt JM Schwartz MA Hirschi KK. Shear-induced Notch-Cx37-p27 axis arrests endothelial cell cycle to enable arterial specification. Nat Commun. 2017;8:2149. doi: 10.1038/s41467-017-01742-7 29247167 10.1038/s41467-017-01742-7 PMC5732288

[48] Luo W Garcia-Gonzalez I Fernández-Chacón M Casquero-Garcia V Sanchez-Muñoz MS Mühleder S Garcia-Ortega L Andrade J Potente M Benedito R. Arterialization requires the timely suppression of cell growth. Nature. 2021;589:437–441. doi: 10.1038/s41586-020-3018-x 33299176 10.1038/s41586-020-3018-x PMC7116692

[49] Zhang H Faber JE. De-novo collateral formation following acute myocardial infarction: dependence on CCR2+bone marrow cells. J Mol Cell Cardiol. 2015;87:4–16. doi: 10.1016/j.yjmcc.2015.07.020 26254180 10.1016/j.yjmcc.2015.07.020 PMC4637183

[50] Anbazhakan S Rios Coronado PE Sy-Quia ANL Seow LW Hands AM Zhao M Dong ML Pfaller MR Amir ZA Raftrey BC. Blood flow modeling reveals improved collateral artery performance during the regenerative period in mammalian hearts. Nature Cardiovascular Research. 2022;1:775–790. doi: 10.1038/s44161-022-00114-9 10.1038/s44161-022-00114-9 PMC10256232 37305211

[51] Yao Y Wang C. Dedifferentiation: inspiration for devising engineering strategies for regenerative medicine. NPJ Regen Med. 2020;5:14. doi: 10.1038/s41536-020-00099-8 32821434 10.1038/s41536-020-00099-8 PMC7395755

[52] Sharma B Chang A Red-Horse K. Coronary artery development: progenitor cells and differentiation pathways. Annu Rev Physiol. 2017;79:1–19. doi: 10.1146/annurev-physiol-022516-033953 27959616 10.1146/annurev-physiol-022516-033953 PMC5513160

[53] Chen HI Sharma B Akerberg BN Numi HJ Kivelä R Saharinen P Aghajanian H McKay AS Bogard PE Chang AH. The sinus venosus contributes to coronary vasculature through VEGFC-stimulated angiogenesis. Development. 2014;141:4500–4512. doi: 10.1242/dev.113639 25377552 10.1242/dev.113639 PMC4302936

[54] Gillich A Zhang F Farmer CG Travaglini KJ Tan SY Gu M Zhou B Feinstein JA Krasnow MA Metzger RJ. Capillary cell-type specialization in the alveolus. Nature. 2020;586:785–789. doi: 10.1038/s41586-020-2822-7 33057196 10.1038/s41586-020-2822-7 PMC7721049

[55] Helker CSM Eberlein J Wilhelm K. Apelin signaling drives vascular endothelial cells towards a pro-angiogenic state. Elife. 2020;9:1–44. doi: 10.7554/ELIFE.55589 10.7554/eLife.55589 PMC7567607 32955436

[56] Tian X Hu T He L Zhang H Huang X Poelmann RE Liu W Yang Z Yan Y Pu WT. Peritruncal coronary endothelial cells contribute to proximal coronary artery stems and their aortic orifices in the mouse heart. PLoS One. 2013;8:e80857. doi: 10.1371/journal.pone.0080857 24278332 10.1371/journal.pone.0080857 PMC3836754

[57] Eyries M Siegfried G Ciumas M Montagne K Agrapart M Lebrin F Soubrier F. Hypoxia-induced apelin expression regulates endothelial cell proliferation and regenerative angiogenesis. Circ Res. 2008;103:432–440. doi: 10.1161/CIRCRESAHA.108.179333 18617693 10.1161/CIRCRESAHA.108.179333

[58] Masoud AG Lin J Azad AK Farhan MA Fischer C Zhu LF Zhang H Sis B Kassiri Z Moore RB. Apelin directs endothelial cell differentiation and vascular repair following immune-mediated injury. J Clin Investig. 2020;130:94–107. doi: 10.1172/JCI128469 31738185 10.1172/JCI128469 PMC6934203

[59] Godoy RS Cook DP Cober ND. Novel apelin-expressing gCap endothelial stem-like cells orchestrate lung microvascular repair. bioRxiv. 2022;2021.07.12.452061. doi: 10.1101/2021.07.12.452061

[60] Mirza MK Sun Y Zhao YD S.K. Potula HH Frey RS Vogel SM Malik AB Zhao YY. FoxM1 regulates re-annealing of endothelial adherens junctions through transcriptional control of β-catenin expression. J Exp Med. 2010;207:1675–1685. doi: 10.1084/jem.20091857 20660612 10.1084/jem.20091857 PMC2916140

[61] Zhao YY Gao XP Zhao YD Mirza MK Frey RS Kalinichenko VV Wang I-C Costa RH Malik AB. Endothelial cell-restricted disruption of FoxM1 impairs endothelial repair following LPS-induced vascular injury. J Clin Investig. 2006;116:2333–2343. doi: 10.1172/jci27154 16955137 10.1172/JCI27154 PMC1555637

[62] He L Liu Q Hu T Huang X Zhang H Tian X Yan Y Wang L Huang Y Miquerol L. Genetic lineage tracing discloses arteriogenesis as the main mechanism for collateral growth in the mouse heart. Cardiovasc Res. 2016;109:419–430. doi: 10.1093/cvr/cvw005 26768261 10.1093/cvr/cvw005 PMC4752045

[63] Zhou D Liu T Wang S He W Qian W Luo G. Effects of IL-1β and TNF-α on the expression of P311 in vascular endothelial cells and wound healing in mice. Front Physiol. 2020;11:545008. doi: 10.3389/fphys.2020.545008 33329015 10.3389/fphys.2020.545008 PMC7729022

[64] Wu B Zhang Z Lui W Chen X Wang Y Chamberlain AA Moreno-Rodriguez RA Markwald RR O'Rourke BP Sharp DJ. Endocardial cells form the coronary arteries by angiogenesis through myocardial-endocardial VEGF signaling. Cell. 2012;151:1083–1096. doi: 10.1016/j.cell.2012.10.023 23178125 10.1016/j.cell.2012.10.023 PMC3508471

[65] Koh BI Lee HJ Kwak PA Yang MJ Kim JH Kim HS Koh GY Kim I. VEGFR2 signaling drives meningeal vascular regeneration upon head injury. Nat Commun. 2020;11:3866. doi: 10.1038/s41467-020-17545-2 32737287 10.1038/s41467-020-17545-2 PMC7395111

[66] Isner JM Pieczek A Schainfeld R Blair R Haley L Asahara T Rosenfield K Razvi S Walsh K Symes JF. Clinical evidence of angiogenesis after arterial gene transfer of phVEGF165 in patient with ischaemic limb. Lancet. 1996;348:370–374. doi: 10.1016/s0140-6736(96)03361-2 8709735 10.1016/s0140-6736(96)03361-2

[67] Kikuchi K Holdway JE Werdich AA Anderson RM Fang Y Egnaczyk GF Evans T Macrae CA Stainier DYR Poss KD. Primary contribution to zebrafish heart regeneration by gata4+ cardiomyocytes. Nature. 2010;464:601–605. doi: 10.1038/nature08804 20336144 10.1038/nature08804 PMC3040215

[68] Jopling C Sleep E Raya M Martí M Raya A Belmonte JCI. Zebrafish heart regeneration occurs by cardiomyocyte dedifferentiation and proliferation. Nature. 2010;464:606–609. doi: 10.1038/nature08899 20336145 10.1038/nature08899 PMC2846535

[69] Bertozzi A Wu CC Hans S Brand M Weidinger G. Wnt/β-catenin signaling acts cell-autonomously to promote cardiomyocyte regeneration in the zebrafish heart. Dev Biol. 2022;481:226–237. doi: 10.1016/j.ydbio.2021.11.001 34748730 10.1016/j.ydbio.2021.11.001

[70] Zhang Y Gago-Lopez N Li N Zhang Z Alver N Liu Y Martinson AM Mehri A MacLellan WR. Single-cell imaging and transcriptomic analyses of endogenous cardiomyocyte dedifferentiation and cycling. Cell Discov. 2019;5:30. doi: 10.1038/s41421-019-0095-9 31231540 10.1038/s41421-019-0095-9 PMC6547664

[71] Lowe V Wisniewski L Pellet-Many C. The zebrafish cardiac endothelial cell—roles in development and regeneration. J Cardiovasc Dev Dis. 2021;8:49. doi: 10.3390/jcdd8050049 34062899 10.3390/jcdd8050049 PMC8147271

[72] Fernández-Chacón M García-González I Mühleder S Benedito R. Role of Notch in endothelial biology. Angiogenesis. 2021;24:237–250. doi: 10.1007/s10456-021-09793-7 34050878 10.1007/s10456-021-09793-7

[73] D’Amato G Phansalkar R Naftaly JA Fan X Amir ZA Rios Coronado PE Cowley DO Quinn KE Sharma B Caron KM. Endocardium-to-coronary artery differentiation during heart development and regeneration involves sequential roles of Bmp2 and Cxcl12/Cxcr4. Dev Cell. 2022;57:2517–2532.e6. doi: 10.1016/j.devcel.2022.10.007 36347256 10.1016/j.devcel.2022.10.007 PMC9833645

[74] Ivins S Chappell J Vernay B Suntharalingham J Martineau A Mohun TJ Scambler PJ. The CXCL12/CXCR4 axis plays a critical role in coronary artery development. Dev Cell. 2015;33:455–468. doi: 10.1016/j.devcel.2015.03.026 26017770 10.1016/j.devcel.2015.03.026 PMC4448146

[75] Cavallero S Shen H Yi C Lien CL Kumar SR Sucov HM. CXCL12 signaling is essential for maturation of the ventricular coronary endothelial plexus and establishment of functional coronary circulation. Dev Cell. 2015;33:469–477. doi: 10.1016/j.devcel.2015.03.018 26017771 10.1016/j.devcel.2015.03.018 PMC4448078

[76] Chang AH Raftrey BC D’Amato G Surya VN Poduri A Chen HI Goldstone AB Woo J Fuller GG Dunn AR. DACH1 stimulates shear stress-guided endothelial cell migration and coronary artery growth through the CXCL12-CXCR4 signaling axis. Genes Dev. 2017;31:1308–1324. doi: 10.1101/gad.301549.117 28779009 10.1101/gad.301549.117 PMC5580653

[77] Marín-Juez R Marass M Gauvrit S Rossi A Lai SL Materna SC Black BL Stainier DYR. Fast revascularization of the injured area is essential to support zebrafish heart regeneration. Proc Natl Acad Sci USA. 2016;113:11237–11242. doi: 10.1073/pnas.1605431113 27647901 10.1073/pnas.1605431113 PMC5056108

[78] Evans CE Iruela-Arispe ML Zhao YY. Mechanisms of endothelial regeneration and vascular repair and their application to regenerative medicine. Am J Pathol. 2021;191:52–65. doi: 10.1016/j.ajpath.2020.10.001 33069720 10.1016/j.ajpath.2020.10.001 PMC7560161

[79] Red-Horse K Ueno H Weissman IL Krasnow MA. Coronary arteries form by developmental reprogramming of venous cells. Nature. 2010;464:549–553. doi: 10.1038/nature08873 20336138 10.1038/nature08873 PMC2924433

[80] Rhee S Chung JI King DA D'amato G Paik DT Duan A Chang A Nagelberg D Sharma B Jeong Y. Endothelial deletion of Ino80 disrupts coronary angiogenesis and causes congenital heart disease. Nat Commun. 2018;9:368. doi: 10.1038/s41467-017-02796-3 29371594 10.1038/s41467-017-02796-3 PMC5785521

[81] Lange M Ohnesorge N Hoffmann D Rocha SF Benedito R Siekmann AF. Zebrafish mutants in vegfab can affect endothelial cell proliferation without altering ERK phosphorylation and are phenocopied by loss of PI3K signaling. Dev Biol. 2022;486:26–43. doi: 10.1016/j.ydbio.2022.03.006 35337795 10.1016/j.ydbio.2022.03.006 PMC11238767

[82] El-Sammak H Yang B Guenther S Chen W Marín-Juez R Stainier DYR. A Vegfc-Emilin2a-Cxcl8a signaling axis required for zebrafish cardiac regeneration. Circ Res. 2022;130:1014–1029. doi: 10.1161/CIRCRESAHA.121.319929 35264012 10.1161/CIRCRESAHA.121.319929 PMC8976759

[83] Marín-Juez R El-Sammak H Helker CSM Kamezaki A Mullapuli ST Bibli S-I Foglia MJ Fleming I Poss KD Stainier DYR. Coronary revascularization during heart regeneration is regulated by epicardial and endocardial cues and forms a scaffold for cardiomyocyte repopulation. Dev Cell. 2019;51:503–515.e4. doi: 10.1016/j.devcel.2019.10.019 31743664 10.1016/j.devcel.2019.10.019 PMC6982407

[84] Giordano FJ Gerber HP Williams SP VanBruggen N Bunting S Ruiz-Lozano P Gu Y Nath AK Huang Y Hickey R. A cardiac myocyte vascular endothelial growth factor paracrine pathway is required to maintain cardiac function. Proc Natl Acad Sci U S A. 2001;98:5780–5785. doi: 10.1073/pnas.091415198 11331753 10.1073/pnas.091415198 PMC33290

[85] Karra R Foglia MJ Choi WY Belliveau C DeBenedittis P Poss KD. Vegfaa instructs cardiac muscle hyperplasia in adult zebrafish. Proc Natl Acad Sci U S A. 2018;115:8805–8810. doi: 10.1073/pnas.1722594115 30104362 10.1073/pnas.1722594115 PMC6126768

[86] Wang Y Zheng Y Zhang W Yu H Lou K Zhang Y Qin Q Zhao B Yang Y Hui R. Polymorphisms of KDR gene are associated with coronary heart disease. J Am Coll Cardiol. 2007;50:760–767. doi: 10.1016/j.jacc.2007.04.074 17707181 10.1016/j.jacc.2007.04.074

[87] Lee KW Lip GYH Blann AD. Plasma angiopoietin-1, angiopoietin-2, angiopoietin receptor Tie-2, and vascular endothelial growth factor levels in acute coronary syndromes. Circulation. 2004;110:2355–2360. doi: 10.1161/01.CIR.0000138112.90641.7F 15302795 10.1161/01.CIR.0000138112.90641.7F

[88] Wada H Suzuki M Matsuda M. VEGF-C and mortality in patients with suspected or known coronary artery disease. J Am Heart Assoc. 2018;7:1–10. doi: 10.1161/JAHA.118.010355 10.1161/JAHA.118.010355 PMC6404168 30554564

[89] Kucukardali Y Aydogdu S Ozmen N Yonem A Solmazgul E Ozyurt M Cingozbay Y Aydogdu A. The relationship between severity of coronary artery disease and plasma level of vascular endothelial growth factor. Cardiovasc Revasc Med. 2008;9:66–70. doi: 10.1016/j.carrev.2007.11.005 18486079 10.1016/j.carrev.2007.11.005

[90] Ohno T Igarashi H Inoue K Akazawa K Joh-o K Hara T. Serum vascular endothelial growth factor: a new predictive indicator for the occurrence of coronary artery lesions in Kawasaki disease. Eur J Pediatr. 2000;159:424–429. doi: 10.1007/s004310051300 10867847 10.1007/s004310051300

[91] Kariyazono H Ohno T Khajoee V Ihara K Kusuhara K Kinukawa N Mizuno Y Hara T. Association of vascular endothelial growth factor (VEGF) and VEGF receptor gene polymorphisms with coronary artery lesions of Kawasaki disease. Pediatr Res. 2004;56:953–959. doi: 10.1203/01.PDR.0000145280.26284.B9 15470196 10.1203/01.PDR.0000145280.26284.B9

[92] Wang Y Huang Q Liu J Wang Y Zheng G Lin L Yu H Tang W Huang Z. Vascular endothelial growth factor A polymorphisms are associated with increased risk of coronary heart disease: a meta-analysis. Oncotarget. 2017;8:30539–30551. doi: 10.18632/oncotarget.15546 28430629 10.18632/oncotarget.15546 PMC5444763

[93] Palmer BR Paterson MA Frampton CM Pilbrow AP Skelton L Pemberton CJ Doughty RN Ellis CJ Troughton RW Richards AM. Vascular endothelial growth factor-A promoter polymorphisms, circulating VEGF-A and survival in acute coronary syndromes. PLoS One. 2021;16:e0254206–e0254215. doi: 10.1371/journal.pone.0254206 34260629 10.1371/journal.pone.0254206 PMC8279389

[94] Rubanyi GM. Mechanistic, technical, and clinical perspectives in therapeutic stimulation of coronary collateral development by angiogenic growth factors. Mol Ther. 2013;21:725–738. doi: 10.1038/mt.2013.13 23403495 10.1038/mt.2013.13 PMC3616542

[95] Kukuła K Chojnowska L Da̧browski M Witkowski A Chmielak Z Skwarek M Kądziela J Teresińska A Małecki M Janik P. Intramyocardial plasmid-encoding human vascular endothelial growth factor A165/basic fibroblast growth factor therapy using percutaneous transcatheter approach in patients with refractory coronary artery disease (VIF-CAD). Am Heart J. 2011;161:581–589. doi: 10.1016/j.ahj.2010.11.023 21392615 10.1016/j.ahj.2010.11.023

