# Supplementary material for: Dedifferentiation and Proliferation of Artery Endothelial Cells Drive Coronary Collateral Development in Mice
Source: Arterioscler Thromb Vasc Biol. 2023 Jun 22;43(8):1455–77. doi: 10.1161/ATVBAHA.123.319319 (PMC10364966; doi:10.1161/ATVBAHA.123.319319)

SUPPLEMENTAL MATERIALS

De-differentiation and Proliferation of Artery Endothelial Cells Drive Coronary Collateral Development in Mice

Gauri Arolkar[1*], Sneha K.[1*], Hanjay Wang[2], Karen M. Gonzalez[3,4], Suraj Kumar[1], Bhavnesh Bishnoi[1], Pamela E. Rios Coronado[5], Y. Joseph Woo[2], Kristy Red-Horse[3,4,6], Soumyashree Das[1#]

[1] National Center for Biological Sciences, Tata Institute of Fundamental Research, Bengaluru, KA 560065, India

[2] Department of Cardiothoracic Surgery, Stanford University School of Medicine, Stanford, CA 94305, USA

[3] Department of Biology, Stanford University, Stanford, CA 94305, USA

[4] Institute for Stem Cell Biology and Regenerative Medicine, Stanford University School of Medicine, Stanford, CA 94305, USA

[5] Department of Bioengineering, Stanford University, Stanford, CA 94305, USA

[6] Howard Hughes Medical Institute

***** Equal contribution

[#] Corresponding author (soumyashree@ncbs.res.in)

Supplemental Material

I. **Figure S1-10 and legends**

II. **Table S1**

III. **Major Resources Table**

IV. **Graphical abstract**

Supplemental Figures and legends

Figure S1: Identification of neonatal *Cadherin5*⁺ cell clusters

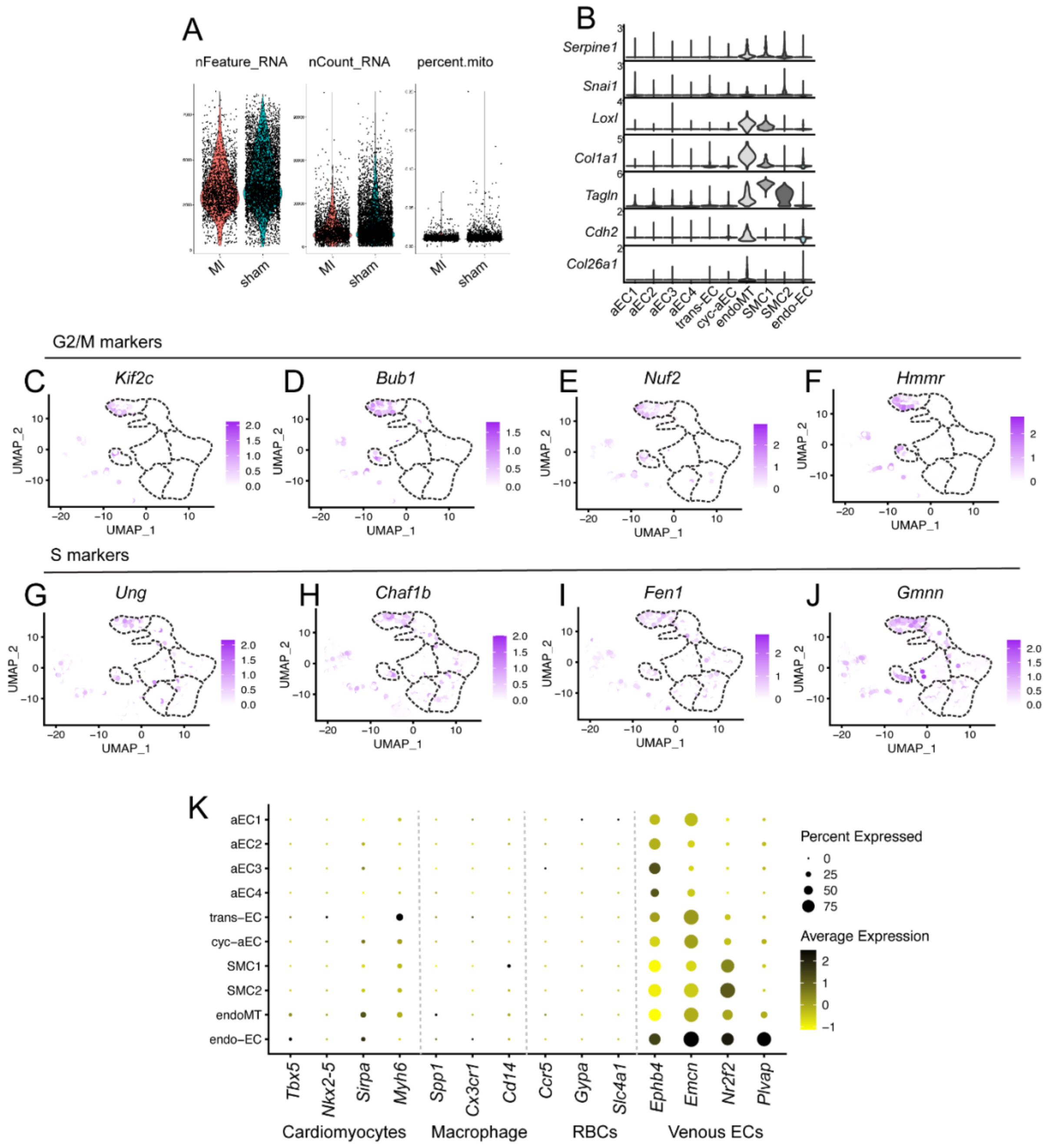

(**A**) Violin plots showing quality control (analyses of nFeature counts, nCount_RNA and mitochondrial genes) performed on neonatal *Cadherin5*$^{+}$ dataset. (**B**) Violin Plot showing expression of endo-MT genes in neonatal *Cadherin5*$^{+}$ cells. (**C**-**F**) Feature plots showing the expression of genes specific to G2/M phase. (**G**-**H**) Feature plots showing the expression of S phase markers in neonatal *Cadherin5*$^{+}$ cells. (**K**) Dot plot showing parentage of cells and average expression of genes, specific to different cell-types.

Figure S2: Artery specific gene expression in neonatal *Cadherin5*⁺ cycling aEC cluster

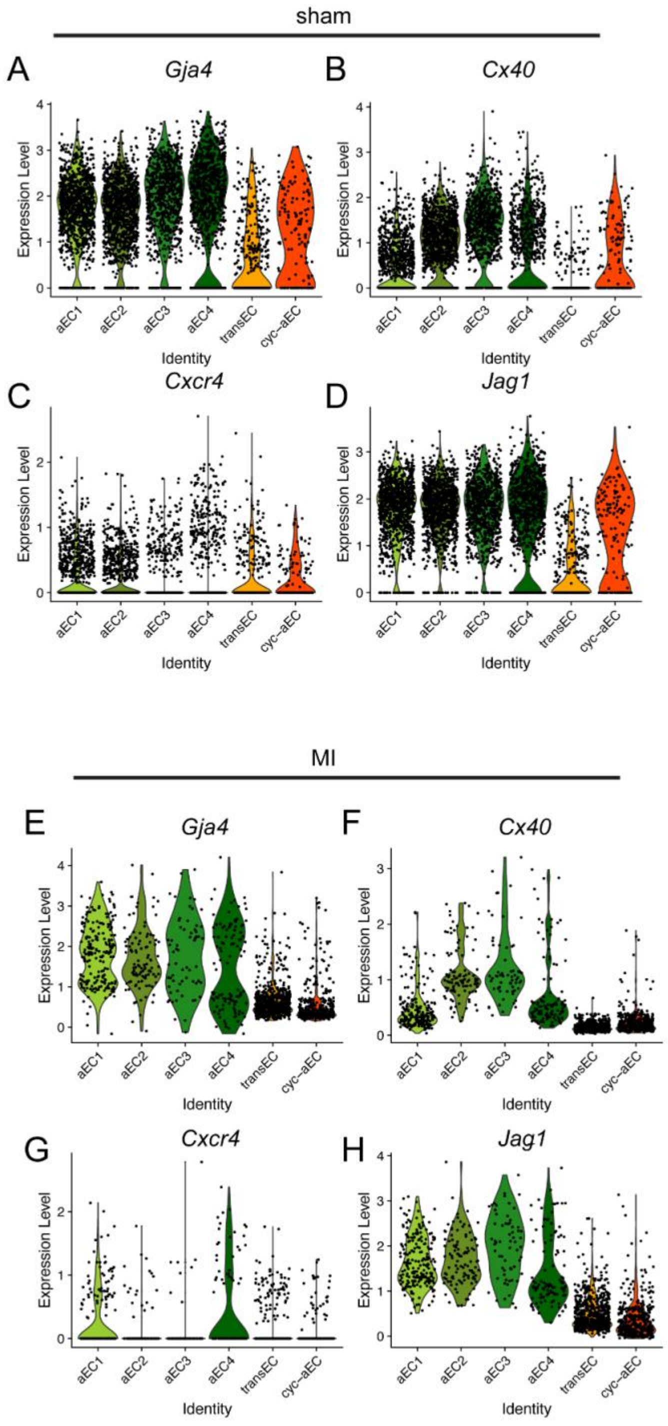

Violin plots showing the expression levels of artery endothelial cell specific markers in *Cadherin5*⁺ neonatal (**A**-**D**) sham and (**E**-**H**) MI cells.

Figure S3: Gene expression analyses of neonatal *Cadherin5*$^{+}$ trans-EC cluster

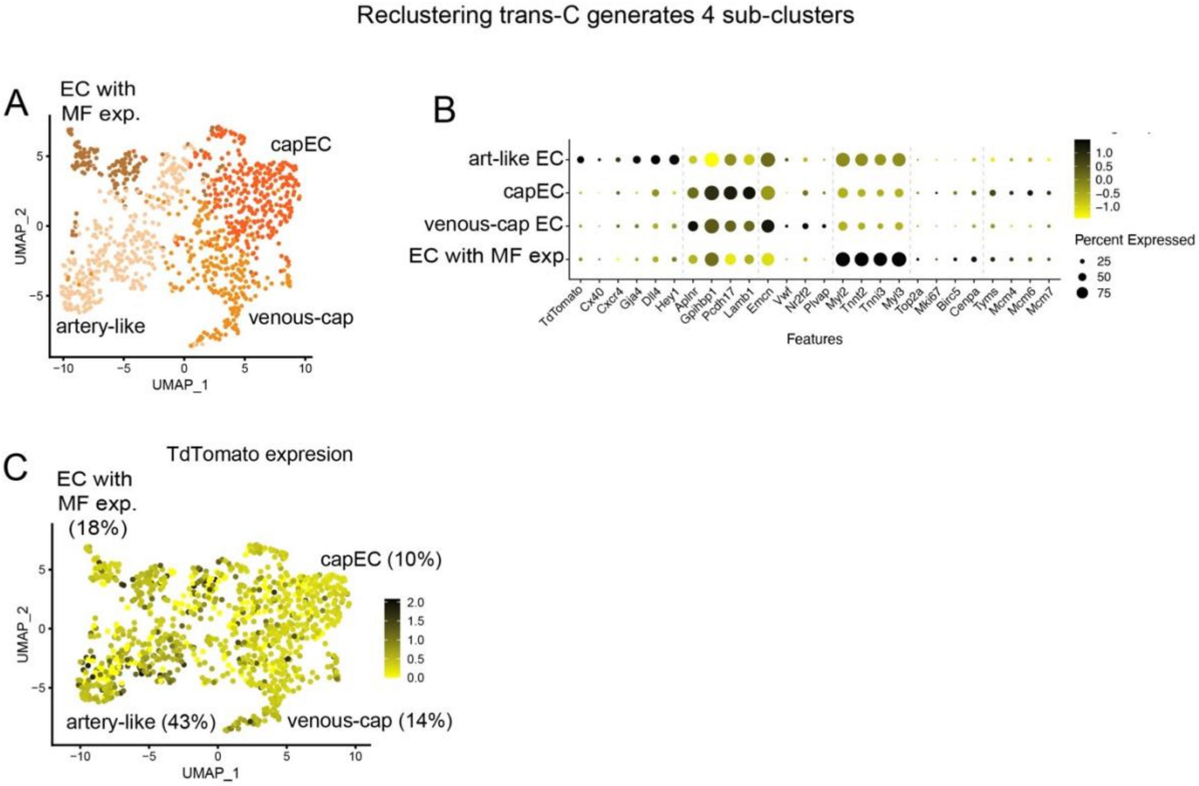

(**A**) Visualization of cells upon re-clustering trans-EC cluster from neonatal dataset, using UMAP. (**B**) Dot plot showing average gene expression and percentage of cells expressing various genes expressed in ECs. (**C**) Feature plot showing distribution of TdTomato expressing cells in **A**. MF, myofibrillar; cap, capillary; exp, expression.

Figure S4: Analyses of post-MI artery EC proliferation in neonatal hearts

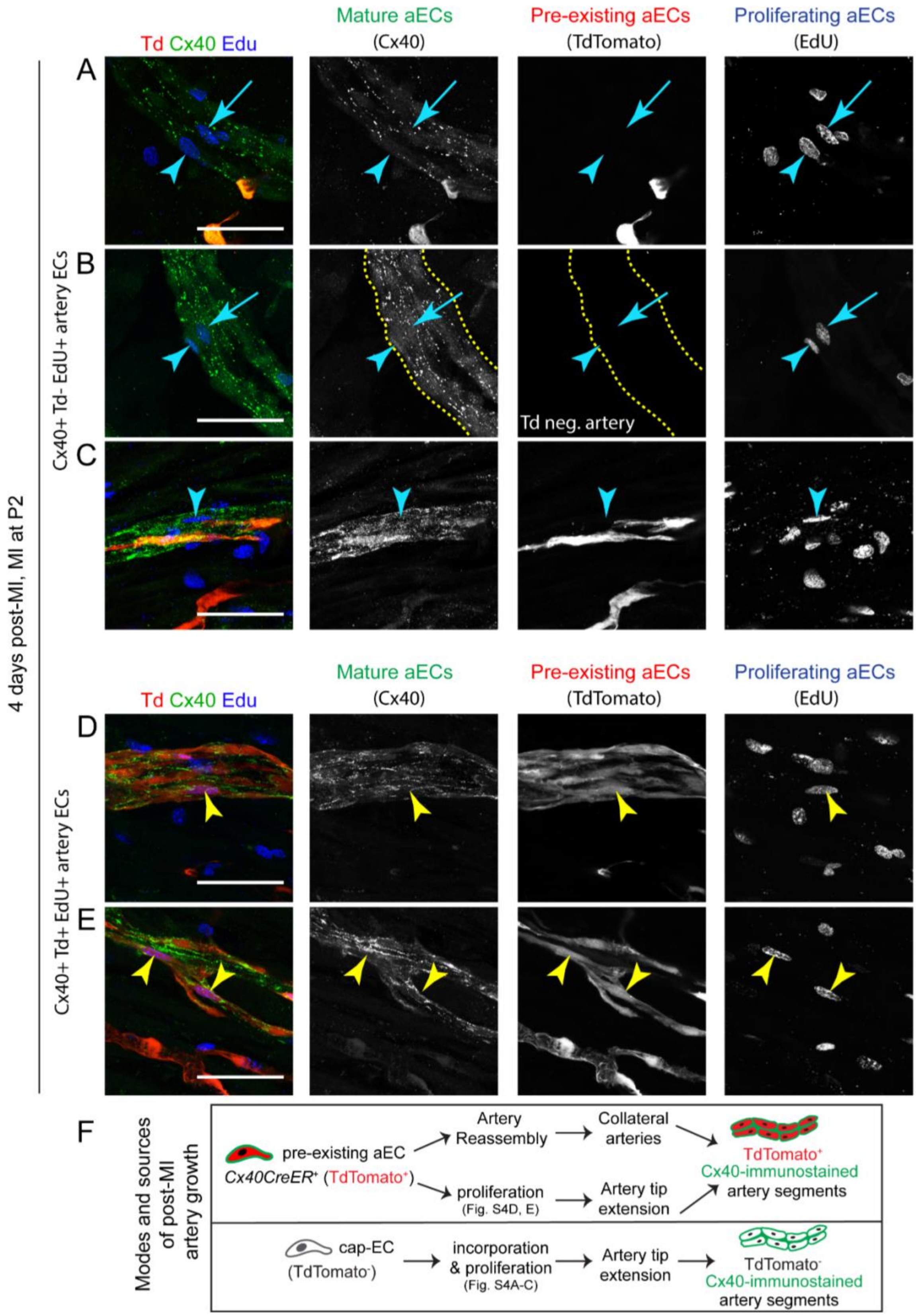

Representative confocal images of artery segments from neonatal P6 hearts, 4 days post MI, immunostained for Cx40, lineage traced for *Cx40creER* (with TdTomato) and labelled for EdU. Images are taken from ischemic regions (watershed area below the ligation). Arrows and arrowheads show (**A**-**C**) EdU^{+} $Cx40^{+}$ $Tdtomato^{-}$ artery ECs and (**D**, **E**) EdU^{+} $Cx40^{+}$ $Tdtomato^{+}$ artery ECs. (**F**) Modes and sources of post-MI artery growth in neonatal hearts, post-MI. neg., negative; aEC, artery endothelial cells; cap-EC, capillary endothelial cells. Td, TdTomato. Scale bar: 50μm

Figure S5: Identification of adult cardiac cell clusters

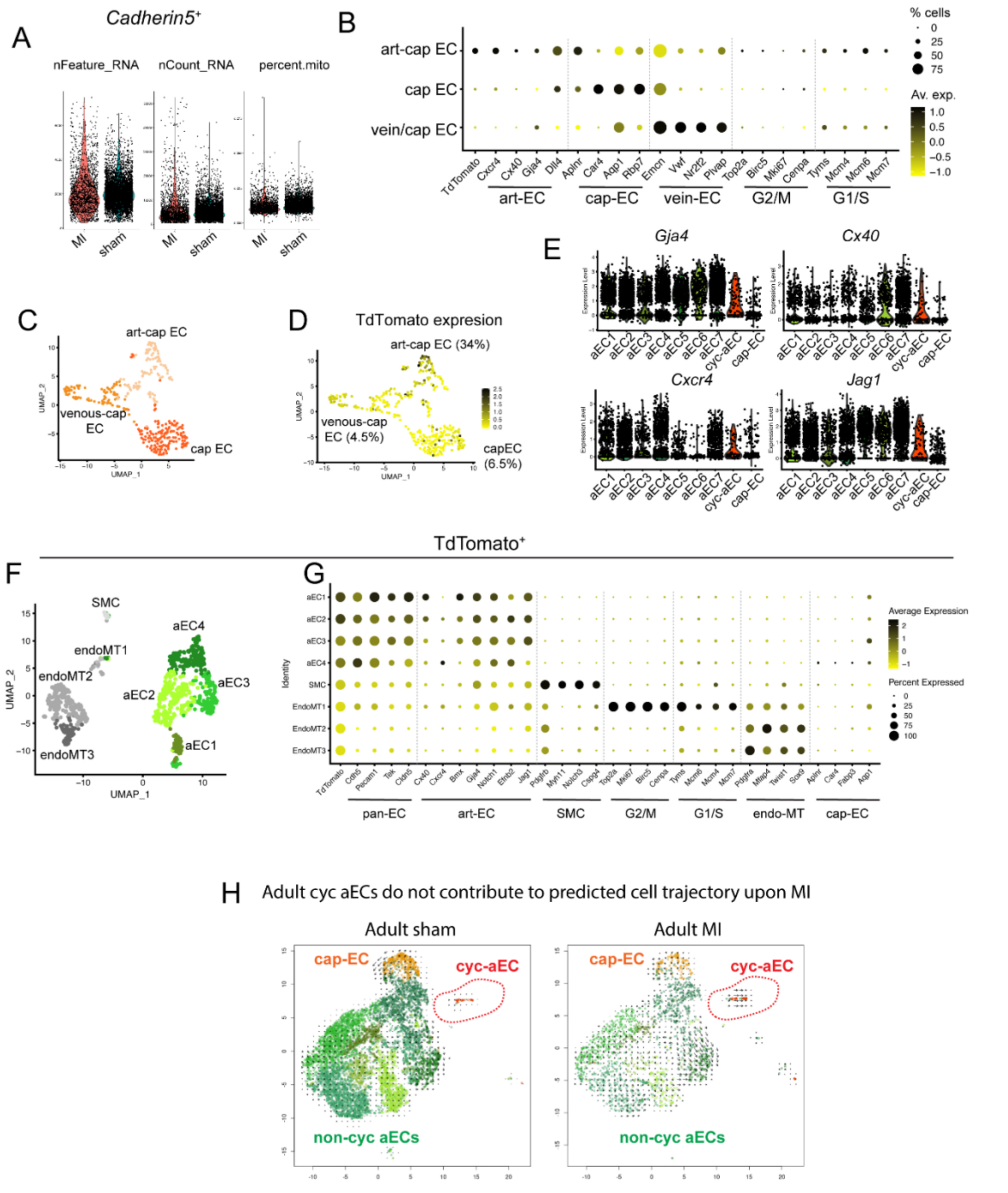

(**A**) Violin plots showing quality control (analyses of nFeature counts, nCount_RNA and mitochondrial genes) performed on adult *Cadherin5*+ dataset. (**B**) DotPlot showing percentage of cells and their average gene expression used for identifying clusters, obtained from re-clustering *Cadherin5*+ cap-ECs. (**C**) Visualization of re-clustered adult *Cadherin5*+ cap-ECs on a UMAP. Clusters were identified using the

DotPlot shown in **B**. (**D**) FeaturePlot showing the distribution of TdTomato expressing cells on re-clustered cap-ECs in **C**. (**E**) Violin Plots showing expression of artery specific genes in adult *Cadherin5*$^{+}$ cells in both sham and MI group, integrated. (**F**) Visualization of clusters in adult TdTomato^{+} cells, using UMAP. (**G**) DotPlot showing average gene expression and percentage of cells expressing cell type specific genes in TdTomato^{+} dataset. (**H**) RNA Velocity analyses of adult *Cadherin5*$^{+}$ cells in sham and MI group.

Figure S6: Analyses of de-differentiation features in *Cadherin5*⁺ neonatal cells

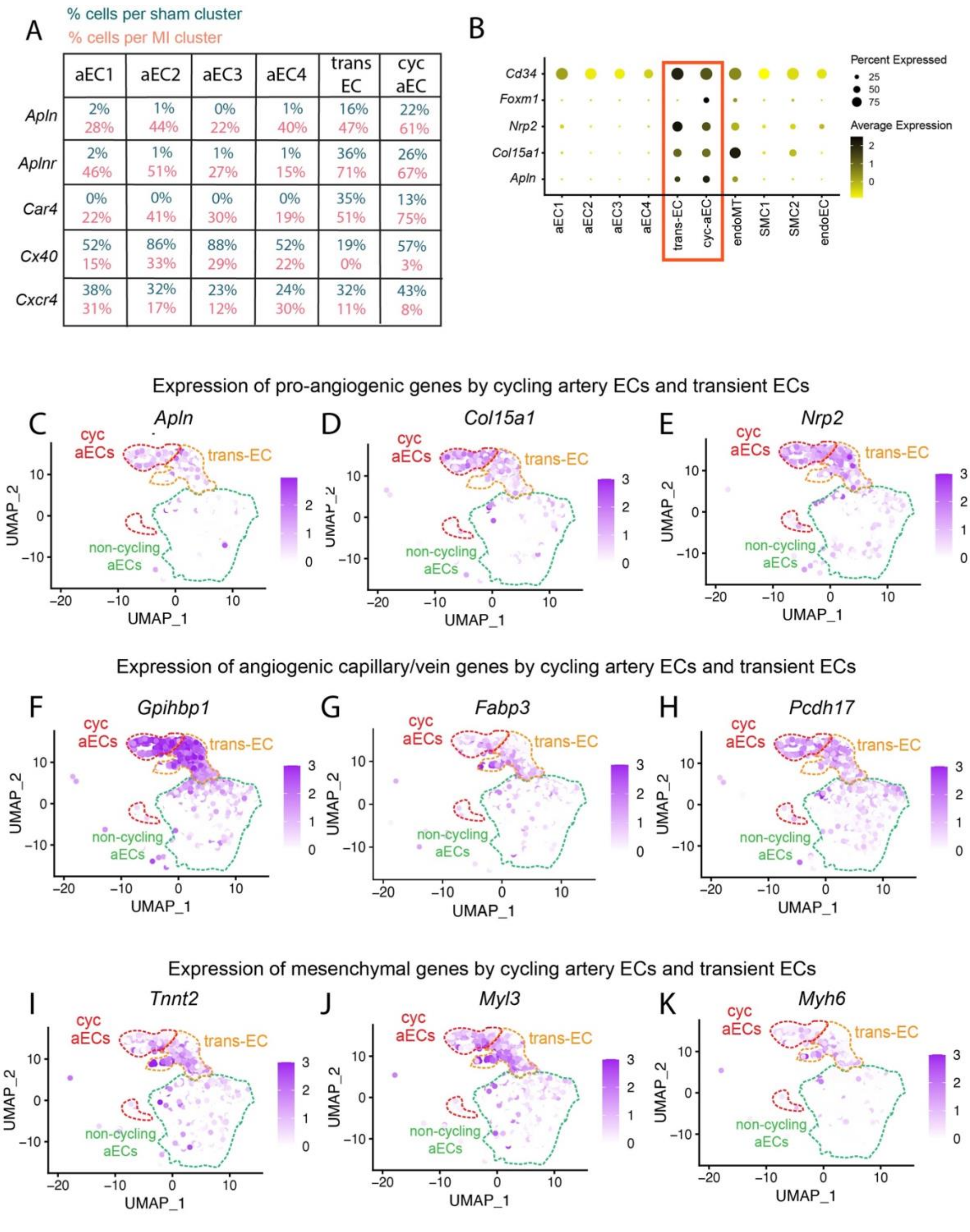

% cells per sham cluster / % cells per MI cluster

	aEC1	aEC2	aEC3	aEC4	trans EC	cyc aEC
Apln	2% 28%	1% 44%	0% 22%	1% 40%	16% 47%	22% 61%
Aplnr	2% 46%	1% 51%	1% 27%	1% 15%	36% 71%	26% 67%
Car4	0% 22%	0% 41%	0% 30%	0% 19%	35% 51%	13% 75%
Cx40	52% 15%	86% 33%	88% 29%	52% 22%	19% 0%	57% 3%
Cxcr4	38% 31%	32% 17%	23% 12%	24% 30%	32% 11%	43% 8%

(**A**) Table showing percentage of sham and MI cells expressing *Apln*, *Aplnr*, *Car4*, *Cx40* and *Cxcr4*. (**B**) DotPlot from neonatal *Cadherin5*⁺ cells showing average expression and percentage of cells expressing pro-angiogenic genes (*Cd34*, *Foxm1*,

Nrp2, *Col15a1*, *Apln*). (**C**-**E**) Feature Plots showing expression of pro-angiogenic *Apln*, *Col15a1*, and *Nrp2* in *Cadherin5*$^{+}$ neonatal cells, from sham and MI together. (**F**-**H**) Feature Plots showing expression of angiogenic genes, also expressed by capillary or venous ECs (*Gpihbp1*, *Fabp3*, *Pcdh17*), in *Cadherin5*$^{+}$ neonatal cells, from sham and MI together. (**I**-**K**) Feature Plots showing expression of mesenchymal genes (*Tnnt2*, *Myl3*, *Myh6*), in *Cadherin5*$^{+}$ neonatal cells, from sham and MI together.

Figure S7: Comparison of de-differentiation features between neonatal *Cadherin5*$^{+}$ cells in cyc-aEC, trans-EC and aEC1 clusters

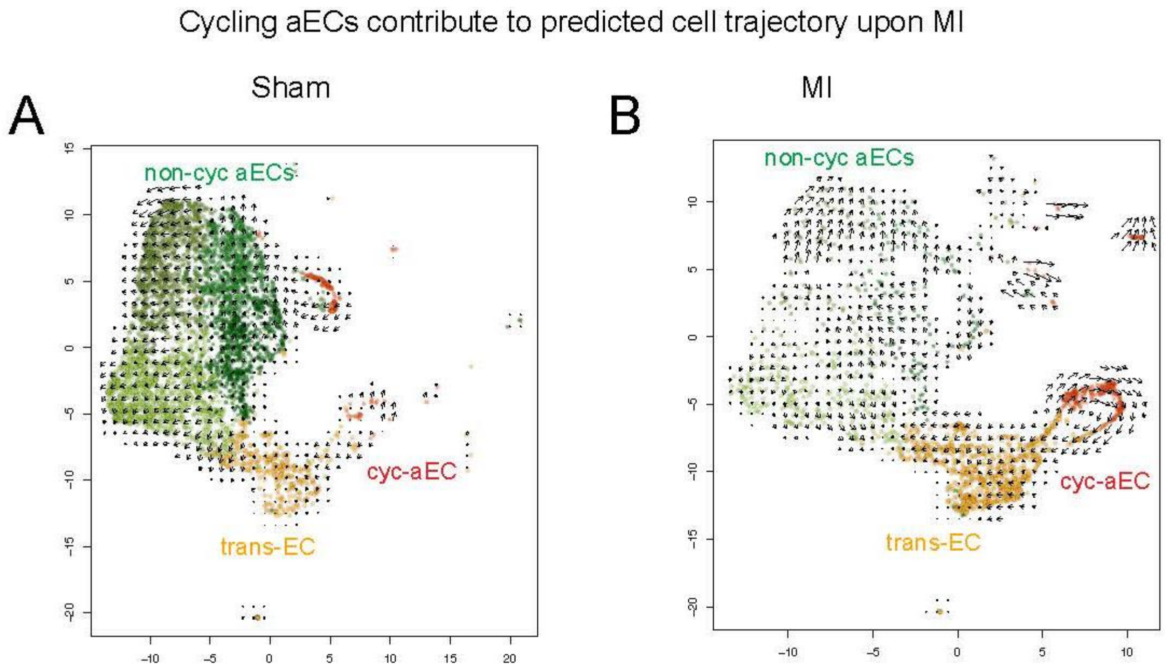

(**A**, **B**) RNA velocity analysis on *Cadherin5*$^{+}$ (**A**) sham and (**B**) MI cells.

Figure S8: Analyses of arterial Vegf pathway in uninjured and injured neonatal hearts

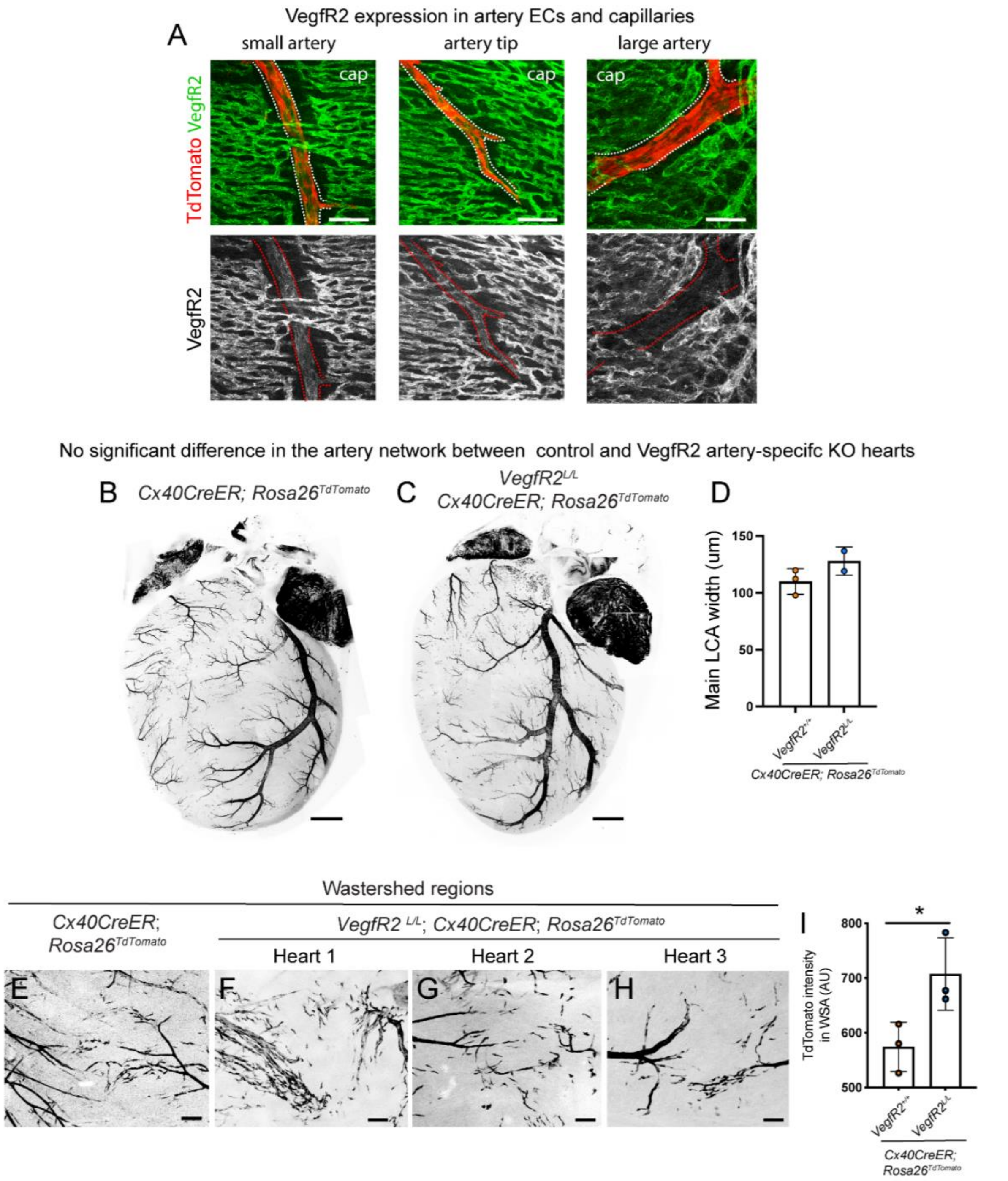

(**A**) Representative confocal images from uninjured P6 *Cx40CreER*; *Rosa26^{TdTomato}* neonatal hearts showing expression of VegfR2 in small arteries, artery tips and capillaries, but not in large arteries. (**B**-**D**) Representative whole heart images from uninjured *Cx40CreER*; *Rosa26^{TdTomato}* mice, (**B**) with or (**C**) without arterial EC

VegfR2. Tamoxifen was injected at P0, hearts were explanted at P6. (**D**) Quantification of width of main left coronary artery (LCA) in uninjured control and hearts deleted for *VegfR2* from arterial ECs. Tamoxifen was injected at P0, hearts were explanted at P6. The difference was statistically insignificant (p-value 0.1881). (**E**) Representative confocal images of wildtype watershed regions from P6 neonatal *Cx40CreER*; *Rosa26*^{TdTomato} hearts, 4 days post-MI. (**F**-**G**) Representative confocal images of *VegfR2* depleted watershed regions from P6 neonatal *Cx40CreER*; *Rosa26*^{TdTomato} hearts, 4 days post-MI. Tamoxifen was administered at P0/P1, MI was performed at P2/P3. **I**) Quantification of *Cx40CreER*-lineage traced (TdTomato^{+}) saECs in wild-type and *VegfR2* depleted watershed regions from P6 neonatal hearts (shown in **E**-**H**), 4 days post-MI. * represents p-value 0.0449. cap, capillary; KO, knockout; wsa, watershed area. Scale bars: **A**: 50µm; **B**, **C**: 500 µm; **E**-**G**: 100 µm

Figure S9: Proliferation status of artery ECs in absence of *VegfR2*

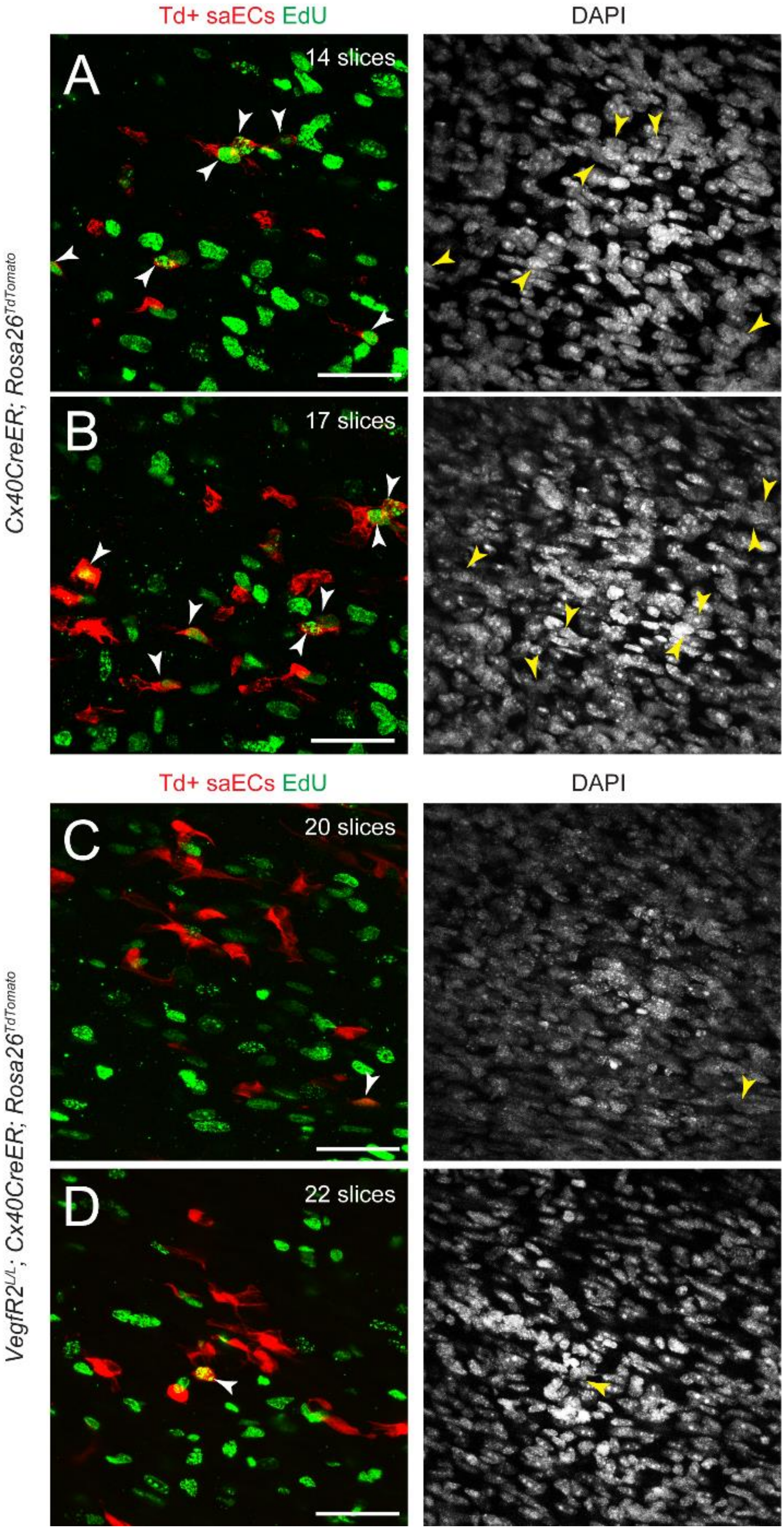

Representative confocal images of EdU^{+} $Tdtomato^{+}$ saECs (white and yellow arrowheads) in watershed regions from *Cx40CreER*-lineage traced hearts, (**A**, **B**) with or (**C**, **D**) without arterial *VegfR2*, 4 days post-MI. Tamoxifen at P0 and MI at P2. Scale bar: 50µm. Td, TdTomato; saECs, single artery endothelial cells

Figure S10: Proposed working model

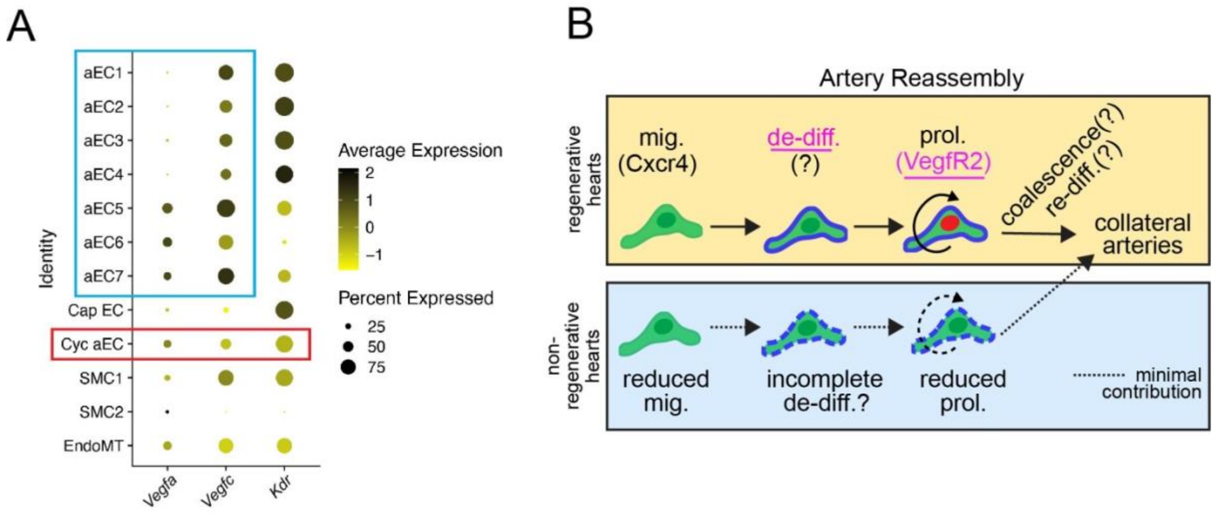

(**A**) Dot plot showing average gene expression and percentage of cells expressing *Vegfa*, *Vegfc* and *VegfR2 (Kdr)* in adult *Cadherin5*$^{+}$ dataset. Blue box indicates ligand expression by aECs, red box indicate downregulation of *VegfR2* in cyc-aEC cluster. (**B**) Proposed working model of cellular events associated with Artery Reassembly. In regenerative neonatal hearts, MI induces sequential migration (via Cxcr4), de-differentiation, and proliferation (via VegfR2) of pre-existing artery ECs, which, leads to formation of collateral arteries and efficient revascularization of ischemic heart regions. Non-regenerative hearts show minimal migration, de-differentiation or proliferation. While Cxcl12/Cxcr4 pathway drives migration, in this study we show that artery EC proliferation is regulated by Vegf pathway. Green, artery ECs; Blue, de-diff (de-differentiation); Red, prol. (proliferation); mig, migration; ? unknown molecules associated with the cellular events linked to Artery Reassembly. Findings of this study (de-differentiation and VegfR2-regulated proliferation of artery ECs) are underlined.

Table S1: Summary of all p-values obtained from Wilcoxon sum rank test

**Statistical significance of gene expression variation as observed using Wilcoxon test**						
**Statistical analysis of de-differentiation genes across neonatal *Cadherin5*$^{+}$ sham and MI cyc-aEC clusters**						
	p_val	auc	avg_log2FC	pct.1	pct.2	p_val_adj
Gja5	6.80E-52	0.225561	-1.39143	0.026	0.569	1.30E-47
Cxcr4	1.71E-20	0.329778	-0.30521	0.082	0.431	3.27E-16
Aplnr	1.75E-17	0.727798	0.699507	0.673	0.257	3.36E-13
Car4	5.90E-06	0.598174	0.413379	0.312	0.125	1.13E-01
Apln	1.57E-14	0.701489	0.762624	0.608	0.222	3.02E-10
Foxm1	No significant difference					
**Statistical analysis of de-differentiation genes across neonatal *Cadherin5*$^{+}$ MI cyc-aEC and trans-EC clusters**						
	p_val	auc	avg_log2FC	pct.1	pct.2	p_val_adj
Gja5	No significant difference					
Cxcr4	No significant difference					
Aplnr	4.82E-12	0.377149	-0.5043	0.673	0.758	9.23E-08
Car4	1.02E-07	0.415964	-0.55117	0.312	0.436	1.95E-03
Apln	No significant difference					

Foxm1	7.04E-44	0.654272	0.367115	0.341	0.031	1.35E-39
**Statistical analysis of de-differentiation genes across neonatal *Cadherin5*$^{+}$ MI trans-EC and aEC1 clusters**						
	p_val	auc	avg_log2FC	pct.1	pct.2	p_val_adj
Gja5	1.64E-24	0.423338	-0.30514	0.001	0.155	3.14E-20
Cxcr4	5.20E-12	0.398321	-0.40858	0.117	0.314	9.97E-08
Aplnr	2.26E-16	0.684601	0.517376	0.758	0.367	4.33E-12
Car4	1.43E-08	0.613955	0.616043	0.436	0.222	2.74E-04
Apln	7.20E-22	0.694867	0.94641	0.488	0.106	1.38E-17
Foxm1	No significant difference					
**Statistical analysis of de-differentiation genes across neonatal *Cadherin5*$^{+}$ MI cyc-aEC and aEC1 clusters**						
	p_val	auc	avg_log2FC	pct.1	pct.2	p_val_adj
Gja5	No significant difference					
Cxcr4	1.00E-14	0.378972	-0.48813	0.082	0.314	1.92E-10
Aplnr	No significant difference					
Car4	No significant difference					
Apln	5.76E-30	0.753943	1.028363	0.608	0.106	1.10E-25
Foxm1	2.78E-19	0.663357	0.399056	0.341	0.014	5.33E-15

**Statistical analysis of de-differentiation genes across adult *Cadherin5*⁺ sham and MI cyc-aEC clusters**						
	p_val	auc	avg_log2FC	pct.1	pct.2	p_val_adj
Gja5	No significant difference					
Cxcr4	0.037701	0.373907	-0.57171	0.327	0.5	1
Aplnr	0.015657	0.612245	0.824505	0.265	0.036	1
Car4	No significant difference					
Apln	0.091189	0.565233	0.623217	0.163	0.036	1
Foxm1	No significant difference					
**Statistical analysis of *Vegf* pathway genes across neonatal *Cadherin5*⁺ sham and MI cyc-aEC clusters**						
	p_val	auc	avg_log2FC	pct.1	pct.2	p_val_adj
Kdr	3.49E-22	0.770358	0.799244	0.933	0.743	6.69E-18
Vegfa	1.53E-21	0.299646	-0.49758	0.144	0.528	2.93E-17
Vegfc	1.81E-51	0.176933	-1.4309	0.099	0.708	3.48E-47
**Statistical analysis of Vegf pathway genes across adult *Cadherin5*⁺ sham and MI cyc-aEC clusters**						
	p_val	auc	avg_log2FC	pct.1	pct.2	p_val_adj
Kdr	1.45E-15	0.125061	-1.96031	0.857	0.97	2.67E-11

Vegfa	3.01E-03	0.586006	0.375123	0.286	0.107	1.00E+00
Vegfc	1.29E-02	0.586856	0.265401	0.388	0.19	1.00E+00

Major Resources Table

Animals (in vivo studies)

Species	Vendor or Source	Background Strain	Sex	Persistent ID / URL
Cx40CreER mouse	Dr. Lucile Miquerol's Lab, IBDM, France.	C57Bl/6	M/F	https://doi.org/10.1002/dvg.20687
Rosa26^{TdTomato} mouse	Jackson Laboratory.	C57BL/6J	M/F	https://www.jax.org/strain/007909
Cx40$^{eGFP/+}$ mouse	Dr. Lucile Miquerol's Lab, IBDM, France.	CD1/129Sv	M/F	https://doi.org/10.1016/j.cardiores.2004.03.007
VegfR2 flox mouse	Jackson Laboratory.	N/A	M/F	https://www.jax.org/strain/018977
ApjCreER mouse	Dr. Kristy Red-Horse's Lab, Department of Biology, Stanford University.	N/A	M/F	https://doi.org/10.1242/dev.113639

Antibodies

Target antigen	Vendor or Source	Catalog #	Dilution Ratio	Working concentration	Lot # (preferred but not required)	Persistent ID / URL
Cx40	Alpha Diagnostics Int. Inc.	CX40-A	1:500	2-20ug/mL	N/A	https://www.4adi.com/4adi/anti-mouse-connexin-40-cx40-igg-1-aff-pure-10947-p.html
VegfR2	R&D Systems	AF644	1:125	5-15ug/mL	N/A	https://www.rndsystems.com/products/mouse-vegfr2-kdr-flk-1-antibody_af644#product-datasheets
Ter119-(Alexafluor-647)	Biolegend	116218	1:100	≤ 0.25 µg per million cells in 100 µl	N/A	https://www.biolegend.com/en-us/products/alexa-fluor-647-anti-mouse-ter-119-erythroid-cells-antibody-

				volume		3277?GroupID=ImportedGROUP1
Rabbit Immunoglobins (Alexaflu or-488, 555)	Invitrogen	A21206, A31572	1:250	N/A	N/A	https://www.thermofisher.com/antibody/product/Donkey-anti-Rabbit-IgG-H-L-Highly-Cross-Adsorbed-Secondary-Antibody-Polyclonal/A-21206 https://www.thermofisher.com/antibody/product/Donkey-anti-Rabbit-IgG-H-L-Highly-Cross-Adsorbed-Secondary-Antibody-Polyclonal/A-31572
Goat Immunoglobins (Alexaflu or-488, 633)	Invitrogen	A11055, A-21082	1:250	N/A	N/A	https://www.thermofisher.com/antibody/product/Donkey-anti-Goat-IgG-H-L-Cross-Adsorbed-Secondary-Antibody-Polyclonal/A-11055 https://www.thermofisher.com/antibody/product/Donkey-anti-Goat-IgG-H-L-Cross-Adsorbed-Secondary-Antibody-Polyclonal/A-21082

Data & Code Availability

Description	Source / Repository	Persistent ID / URL
Single Cell RNA sequencing data, raw sequence files and Cell Ranger processed data	Gene Expression Omnibus	accession ID: GSE210307
Scripts used for the Single Cell RNA sequencing data analysis	GitHub	https://github.com/Snehasrivatsa/scRNA

Other

Description	Source / Repository	Persistent ID / URL

Collagenase	Worthington (#LS004186)	https://www.worthington-biochem.com/products/collagenase
Dispase	Worthington (#LS02100)	https://www.worthington-biochem.com/products/neutral-protease-dispase
DNase I	Worthington (#LS002007)	https://www.worthington-biochem.com/products/deoxyribonuclease-i
Tamoxifen	Sigma (#T5648-1G)	https://www.sigmaaldrich.com/IN/en/product/sigma/t5648
Triton-X-100	Sigma (#X100-100ML) ,Qualigens (#Q10655)	https://www.sigmaaldrich.com/IN/en/product/sial/x100<br><br>https://www.thermofisher.in/chemicals/shop/products/triton-x-100-biochemistry-qualigens-2/Q10655
PBS	Thermofisher (#10010023)	https://www.thermofisher.com/order/catalog/product/10010023
5-ethynyl-2'-deoxyuridine (EdU)	Invitrogen (#A10044)	https://www.thermofisher.com/order/catalog/product/A10044
Click-iT staining kit	Invitrogen (#C10340)	https://www.thermofisher.com/order/catalog/product/C10340
Vecta-Shield	VECTOR LAB (#H-1000).	https://vectorlabs.com/products/mounting/vectashield
Glycerol	Merck	https://www.sigmaaldrich.com/IN/en/product/sial/g9012?gclid=CjwKCAjwov6hBhBsEiwAvrvN6OiOaKXEaQjRc-1MtS7RtgQWK1t8L_xsr4zZwz2vjY7xobOtYSebrxoCfCkQAvD_BwE&gclsrc=aw.ds
DAPI	Sigma (#D9542-1mg)	https://www.sigmaaldrich.com/IN/en/search/d9542-1mg?focus=products&page=1&perpage=30&sort=relevance&term=d9542-1mg&type=product_name
Dimpled (microscope) slides	Sail brand, (# 7104)	N/A
Coverslips (0.13-0.17mm)	Thermo Scientific (# 48367-059)	N/A

non-absorbable prolene sutures	Ethicon Ethilon	N/A

ARRIVE GUIDELINES
The ARRIVE guidelines (https://arriveguidelines.org/) are a checklist of recommendations to improve the reporting of research involving animals. Key elements of the study design should be included below to better enable readers to scrutinize the research adequately, evaluate its methodological rigor, and reproduce the methods or findings.

Study Design

1. **Single Cell RNA sequencing.**

Groups	Sex	Age	Number (prior to experiment)	Number (after termination)	Littermates (Yes/No)	Other description
Group 1 (neonate sham)	M/F	P4/P5	15-20	15-20	Yes	
Group 2 (neonate MI)	M/F	P4/P5	15-20	15-20	Yes	
Group 3 (adult sham)	M/F	2-3 months old	10-12	10-12	Yes	
Group 4 (adult MI)	M/F	2-3 months old	10-12	10-12	Yes	

2. **Collateral artery detection in Figure 6 D-J.**

Groups	Sex	Age	Number (prior to experiment)	Number (after termination)	Littermates (Yes/No)	Other description
Group 1 (neonate, no MI)	M/F	P6	3	3	Yes	MI at P2
Group 3 (neonatal MI, wild-type)	M/F	P6/P7	8	8	Yes	MI at P2/P3
Group 4 (neonatal MI, heterozygous for *VegfR2* allele)	M/F	P6/P7	5	5	Yes	MI at P2/P3

Group 5 (neonatal MI, knockout for *VegfR2* allele)	M/F	P6/P7	14	14	Yes	MI at P2/P3
Group 6 (neonatal MI, wild-type)	M/F	P11	3	3	Yes	MI at P7

3. **Proliferation (EdU) status of saECs in neonatal MI hearts with and without *VegfR2* in Figure 6K-M.**

Groups	Sex	Age	Number (prior to experiment)	Number (after termination)	Littermates (Yes/No)	Other description
Group 1 (neonatal MI, wild-type)	M/F	P6/P7	6	6	Yes	MI at P2/P3
Group 2 (neonatal MI-knockout for *VegfR2* allele)	M/F	P6/P7	5	5	Yes	MI at P2/P3
Group 3 (neonatal MI, wild-type)	M/F	P11	3	3	Yes	MI at P7

4. **Cx40 staining in arteries and saECs in neonatal MI hearts in Figure 1N-P and 4C-D.**

Groups	Sex	Age	Number (prior to experiment)	Number (after termination)	Littermates (Yes/No)	Other description
Group 1 (neonatal MI at P2)	M/F	P6	4-5	4-5	Yes	Single artery ECs (4 hearts), artery tips (5 hearts)

5. **Apelin expression in saECs in neonatal MI hearts in Figure 4E-G.**

Groups	Sex	Age	Number (prior to experiment)	Number (after termination)	Littermates (Yes/No)	Other description

Group 1 (neonatal MI)	M/F	P6	3	3	Yes	MI at P2

6. **Cardiac function in adults with and without *VegfR2* in Figure 6N-Q.**

Groups	Sex	Age	Number (prior to experiment)	Number (after termination)	Littermates (Yes/No)	Other description
Group 1 (Control)	M/F	P32/33	27	27	Yes	MI at P2/P3
Group2 (knockout for *VegfR2* allele)	M/F	P32/33	10	10	Yes	MI at P2/P3

7. **VegfR2 immunostaining in uninjured neonatal hearts in Figure S8A**

Groups	Sex	Age	Number (prior to experiment)	Number (after termination)	Littermates (Yes/No)	Other description
Group 1 (neonatal no MI)	M/F	P6	3	3	Yes	

8. **LCA width comparison with and without *VegfR2* in uninjured neonatal hearts in Figure S8B-D.**

Groups	Sex	Age	Number (prior to experiment)	Number (after termination)	Littermates (Yes/No)	Other description
Group 1 (neonatal no MI, wild-type)	M/F	P6	3	3	Yes	
Group2 (neonatal no MI-knockout for *VegfR2* allele)	M/F	P6	2	2	Yes	

9. **Tdtomato Intensity with and without *VegfR2* in MI neonatal hearts in Figure S8E-I.**

Groups	Sex	Age	Number (prior to experiment)	Number (after termination)	Littermates (Yes/No)	Other description
Group 1 (neonatal MI, wild-type)	M/F	P6/P7	3	3	Yes	MI at P2/P3
Group2 (Neonatal MI-knockout for *VegfR2* allele)	M/F	P6/P7	3	3	Yes	MI at P2/P3

Sample Size: Please explain how the sample size was decided Please provide details of any a *prior* sample size calculation, if done.
Not available
Inclusion Criteria
Not available
Exclusion Criteria
Not available
Randomization
Not available
Blinding
Not available

Graphical Abstract

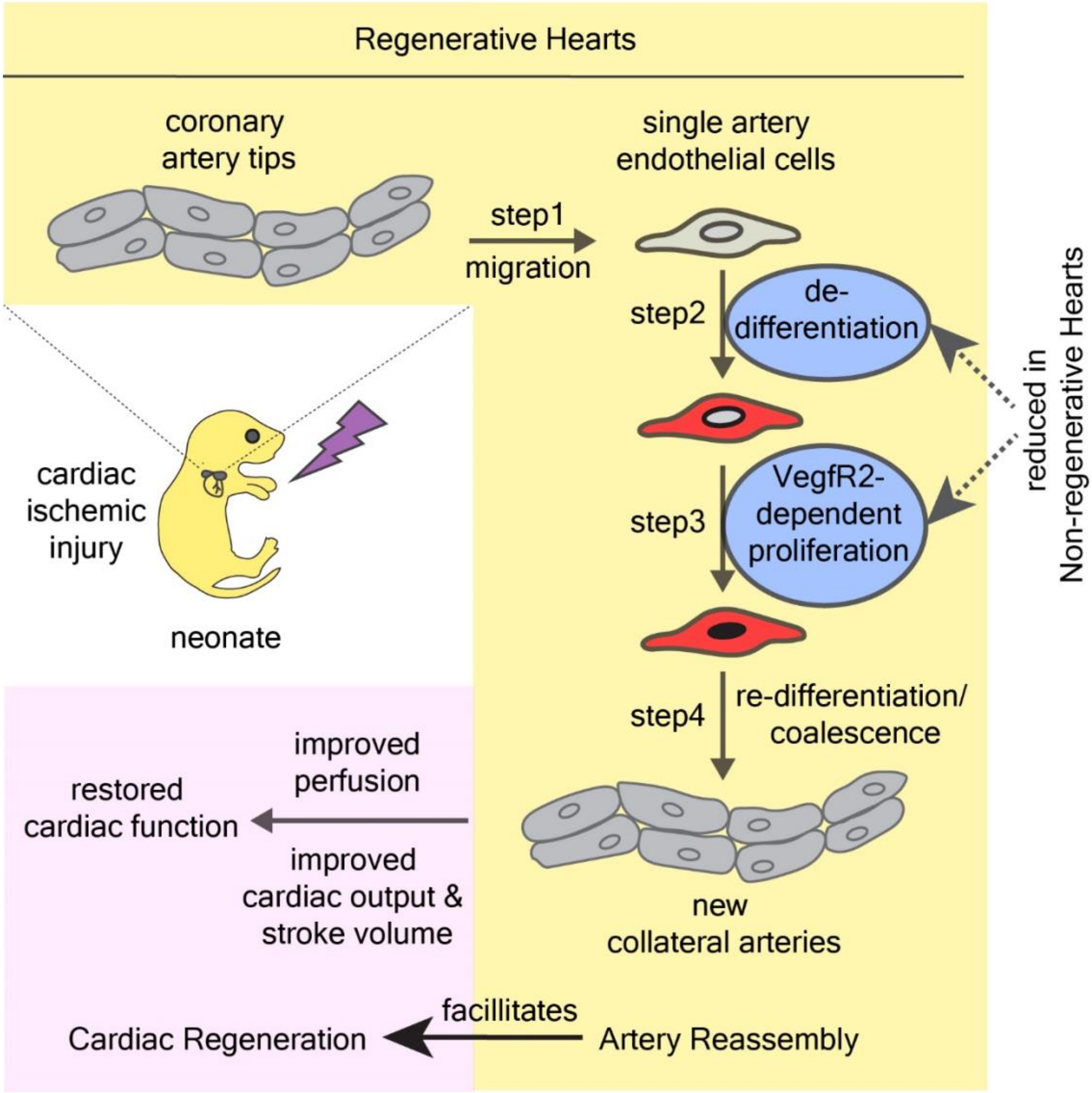

Supplement: Supplementary file 1 [file atv-43-1455-s001.pdf]
